# Non-specific markers of inflammation in bare-nosed wombats (*Vombatus ursinus*) with sarcoptic mange

**DOI:** 10.3389/fvets.2024.1403221

**Published:** 2024-06-26

**Authors:** Vicky Wilkinson, Shane A. Richards, Scott Carver, Christina Næsborg-Nielsen, Carolyn Cray, Gabriele Rossi

**Affiliations:** ^1^School of Natural Sciences, University of Tasmania, Hobart, TAS, Australia; ^2^Division of Comparative Pathology, Department of Pathology and Laboratory Medicine, University of Miami, Miami, FL, United States; ^3^School of Veterinary Medicine, Murdoch University, Perth, WA, Australia

**Keywords:** acute phase response, agarose gel electrophoresis, assay validation, bare-nosed wombat, haptoglobin, erythrocyte sedimentation rate, *Sarcoptes scabiei*, sarcoptic mange

## Abstract

Sarcoptic mange, caused by epidermal infection with *Sarcoptes scabiei*, negatively impacts the health, welfare, and local abundance of bare-nosed wombats (*Vombatus ursinus*) in Australia. Improved understanding of the host immune response to disease and its contribution to pathophysiology could be used to inform management actions for this species *in* and *ex situ*. To evaluate the immune response of bare-nosed wombats to sarcoptic mange, we validated three assays (haptoglobin, agarose gel electrophoresis, and micro-erythrocyte sedimentation rate) measuring non-specific markers of inflammation using serum samples from free-living wombats from Tasmania (*n* = 33). We then analysed correlations between the assay results for each non-specific marker of inflammation and wombat’s sarcoptic mange scores, and performed histopathological examinations to investigate association of the acute phase response with systemic amyloidosis. We present evidence that haptoglobin and erythrocyte sedimentation rate increased, and albumin decreased, in association with sarcoptic mange scores. This research demonstrates links between the acute phase response and sarcoptic mange severity in bare-nosed wombats, highlighting the utility of non-specific markers of inflammation for aiding assessment of the systemic effects of mange. Showing the value of agarose gel electrophoresis, we also identified specific acute phase proteins warranting future evaluation and found evidence of an immunoglobulin response in mange-affected wombats, revealed by increasing γ-globulins in association with apparent disease severity. Meanwhile, owing to its relatively low resource requirements and rapidity, the erythrocyte sedimentation rate assay may be useful as a point-of-care test to support therapeutic decisions in the field. Our methods and findings are likely to be applicable to a range of other clinical and population health scenarios in captive and free-living wombats, and species impacted by sarcoptic mange globally.

## 1 Introduction

Elucidating the immune response of wildlife hosts to pathogens provides valuable insights for *ex-* and *in situ* disease management (1, 2). Sarcoptic mange, caused by epidermal *Sarcoptes scabiei* infection, is a globally significant ectoparasitic disease for which such insights can inform management decisions (3). Recorded in *circa* 150 wild and domestic mammalian species (3), *S. scabiei* burrows into the stratum corneum of the host epidermis, eliciting an immunopathological response that results in disease (4). Clinical signs typically comprise progressive erythema, dermatitis, intense pruritis, hyperkeratosis, alopecia, and weight loss (5). Despite similarities in clinical presentations, disease severity and outcomes vary, ranging from recovery via development of acquired immunity, to mortality through systemic pathophysiological processes, such as disseminated bacterial infections and amyloidosis (2, 4, 6). These differences can occur at inter-individual, −population, and -species levels, and improvements in understanding of the markers and drivers of the different manifestations of sarcoptic mange in captive and free-living wildlife would enhance abilities to make more informed management decisions (1, 2, 7).

As in other wildlife-pathogen systems (8), variation in the type and scale of immune responses may be a determinant of intra- and inter-species differences in mange pathogenesis (1, 2, 9, 10). For example, the activity of immunoglobulins, which are serum proteins produced by inflammatory cells (i.e., plasma cells and B lymphocytes), may provide insights into individual and species-level variation in the severity of mange-associated skin lesions and development of protective immunity (7, 11, 12). Acute phase (AP) proteins, which are produced in the liver and form part of the AP response, are a component of the innate immune system that can also be used to measure immune variation (1). During an AP response to factors such as infection and inflammation, AP proteins undergo increases or decreases in serum concentration (and are therefore termed positive or negative AP proteins, respectively) (1, 9, 10), and are involved in processes that promote health and the restoration of homeostasis, including chemotaxis, immunomodulation, and bacteriostasis (13, 14). Due to their rapid response times (15), changes in AP protein concentrations can be more sensitive biomarkers of inflammation when compared with common clinical indicators such as pyrexia or leukograms (16–18). The scale of changes also often correlates with overall disorder severity (9, 14), and variation in the speed, magnitude, and duration of the response among different AP proteins aids in the interpretation of clinical progression and distinguishing between acute and chronic inflammatory states (19).

Acute phase proteins therefore have the potential to be valuable for use in wildlife, where abilities to diagnose, monitor, treat, and prognosticate are limited by diagnostic test availability and tendencies of wild animals to hide clinical signs (15, 20). Though AP proteins are non-specific markers of inflammation (NSMI) and do not provide a precise diagnosis (18, 20), assays measuring their activity are proving increasingly useful for captive and free-living wildlife health research, to detect sub-clinical and chronic inflammation, assess welfare, monitor disease progression and response to treatment, and indicate prognosis (16, 21, 22). Examples include the high sensitivity of haptoglobin as a diagnostic tool for subclinical *Mycobacteria bovis* infection in red deer (*Cervus elaphus*) and African buffalo (*Syncerus caffer*) (23), and the establishment of AP protein reference intervals in koala (*Phascolarctos cinereus*) to improve the diagnosis and treatment of common conditions such as chlamydiosis and cryptococcosis (15). The scalability of assays has also enabled their application to population health investigations, including: monitoring baseline health (22, 24); augmenting disease surveillance (17); identifying compromised (24) or susceptible groups (8, 25); and, investigating inter-species differences in pathogen tolerance (26). At the population scale, NSMIs therefore represent useful management tools, for example by enabling the identification and removal of Alpine ibex (*Capra ibex*) with asymptomatic *S. scabiei* to reduce the risk of pathogen introduction into naïve populations (9).

For sarcoptic mange, AP responses are likely driven by disease-induced tissue damage and hypersensitivity reactions, invasion of cutaneous lesions by pathogenic microorganisms (27), and immunomodulatory effects of *S. scabiei* that promote AP protein expression (9). Consequently, studies involving wildlife species, comprising Alpine ibex (*Capra ibex*) (9), Iberian ibex (*Capra pyrenaica*) (7), capybara (*Hydrochoerus hydrochaeris*) (16), and dromedary camel (*Camelus dromedaries*) (28), have all demonstrated AP responses in association with sarcoptic mange (Table 1). The value of several positive AP proteins as NSMI is evidenced by increasing concentrations in relation to mange severity in Iberian ibex (1, 7) and decreasing concentrations in response to treatment in capybara (16). However, AP responses can also be detrimental, including damaging internal organs via amyloidosis (1, 30, 31), which has been found to play a role in mange pathophysiology and mortality in Iberian ibex (1). In commonality with other species and disease processes (14, 32, 33), these findings indicate that AP proteins are associated with sarcoptic mange pathogenesis and outcomes, and thus provide a focus for developing tools to aid with investigations into individual, population, and species-level responses to infection with *S. scabiei* (7, 9). Evidence suggests *S. scabiei* emerged in Australia through European colonialism and that the pathogen now occurs throughout the range of the bare-nosed wombat (*Vombatus ursinus*) (34), a nocturnal and semi-fossorial marsupial (35). Analogous to other heavily impacted wildlife species (2, 36), sarcoptic mange in bare-nosed wombats results in high morbidity and mortality rates (35). Though population-level effects of disease have been limited, infrequent epizootics have caused local declines and extirpations, and can have knock-on effects for other species (37–39). Despite being a well-studied host-disease system (35), questions remain regarding the mechanisms underlying high rates of disease-induced mortality, the host immune response to *S. scabiei* infection, and the potential for host-factors to influence epidemiology (5, 6). Disease management interventions currently take the form of reactive pathogen-targeted treatment strategies *in* and *ex situ* (40–42), with health assessments predominantly based on the evaluation of visual clinical signs, for example via sarcoptic mange scoring (35, 40, 41). Nonetheless, there is much to be learned about how external clinical signs relate to the systemic disease state, particularly as changes in haematology and serum biochemistry profiles have proved highly variable in this species (43, 44), including in the sarcoptic mange context (4).

**Table 1:** Published acute phase protein responses to sarcoptic mange in wildlife.

Acute phase protein	Species	(Latin name)	Response	References
Haptoglobin	Alpine ibex	*(Capra ibex)*	Positive*	Rahman et al. (9)
	Capybara	*(Hydrochoerus hydrochaeris)*	Positive*	Bernal et al. (16)
	Iberian ibex	*(Capra pyrenaica)*	Positive	Ráez-Bravo et al. (7)
	Dromedary camel	*(Camelus dromedaries)*	Positive*	Hassan et al. (28)
Serum amyloid A	Alpine ibex	*(Capra ibex)*	Positive*	Rahman et al. (9)
	Iberian ibex	*(Capra pyrenaica)*	Positive	Ráez-Bravo et al. (7)
	Iberian ibex	*(Capra pyrenaica)*	Positive*	Espinosa et al. (1)
	Dromedary camel	*(Camelus dromedaries)*	Positive*	Hassan et al. (28)
α-1-acid glycoprotein	Alpine ibex	*(Capra ibex)*	Positive*	Rahman et al. (9)
	Capybara	*(Hydrochoerus hydrochaeris)*	Positive*	Bernal et al. (16)
	Iberian ibex	*(Capra pyrenaica)*	Positive	Ráez-Bravo et al. (7)
Ceruloplasmin	Alpine ibex	*(Capra ibex)*	Positive*	Rahman et al. (9)
	Dromedary camel	*(Camelus dromedaries)*	Positive*	Hassan et al. (28)
Albumin	Capybara	*(Hydrochoerus hydrochaeris)*	Negative*	Bernal et al. (16)
	Bare-nosed wombat	*(Vombatus ursinus)*	Negative*	Skerratt et al. (29)
Acetylcholinesterase	Iberian ibex	*(Capra pyrenaica)*	Positive*	Espinosa et al. (1)

*Indicates statistically significant difference between sarcoptic mange-affected animals and controls, as determined by overlap assessment.

Greater knowledge of the host immune response to *S. scabiei* infection in wombats, in particular the activity of AP and other serum proteins, therefore has potential to enhance health monitoring (31), support evidence-based management decisions (9), and reinforce guidelines that safeguard wombat welfare. As such, we aim to: (i) conduct analytical and clinical validation of three assays measuring NSMI in bare-nosed wombats; (ii) characterise the relationship between apparent sarcoptic mange severity and NSMI within affected hosts; and (iii), investigate the contribution of the AP response to mange pathogenesis.

## 2 Materials and methods

### 2.1 Study animals and sampling

Twenty-three adult free-living bare-nosed wombats (12 male, 11 female) were opportunistically captured using hand nets from March to September 2021 at Cape Portland, Tasmania, Australia (GPS −40.786, 148.022). Wombats were anaesthetised with intramuscular tiletamine/zolazepam (3 mg/kg) and medetomidine (40 μg/kg) and routinely monitored at five-minute intervals while under anaesthesia. Procedures then included clinical examination, aseptic jugular venipuncture using a 21-gauge needle, and deep skin scrapes of representative flank lesions (45). Additionally, the average mange score was determined for all animals by: dividing the body into seven segments bilaterally (i.e., head, shoulder, forelimb, back, flank, rump, and hindlimb); allocating a score of 0–10 to each segment based on clinical sign severity; and, calculation of the mean score (35). Anaesthesia was reversed with intramuscular atipamezole at 5x the medetomidine dose and animals were housed in insulated PP50 extra-large crates until ambulatory. Euthanasia of captured wombats was based on national guidelines (46) and these animals (*n* = 3) were subjected to the above protocols prior to intracardiac overdose of pentobarbital sodium.

Additional samples were obtained from 12 adult moribund free-living wombats (two male, four female, six unknown) presented to Bonorong Wildlife Sanctuary from locations across Tasmania for euthanasia throughout 2021–2022. These animals were exposed to 5% isoflurane (Henry Schein) at 3 L/min oxygen in an induction chamber until recumbent, then 2–5% isoflurane at 2 L/min oxygen via facemask for maintenance. Blood was collected from the left ventricle using a 21-gauge needle, after which euthanasia, average mange score, and skin scrapes were performed as above. Necropsies were then performed, and representative samples of heart, kidney, liver, lung, skeletal muscle, spleen, skin, gonad, and inguinal lymph node were fixed in 10% neutral buffered formalin. Samples were then: submitted to a private veterinary pathology laboratory (ASAP Laboratories); embedded in paraffin, sectioned, and stained with H&E and Congo Red; and, subjected to routine histopathologic examination to investigate the presence of amyloid material. Sarcoptic mange diagnoses were based on clinical signs, quantified by average mange scores (40), and confirmed by presence or absence of *S. scabiei* in skin scrapes examined under a light microscope, using characteristic morphological features for identification (45). All work was approved by the University of Tasmania’s Animal Ethics Committee (Approval Number: 20370).

### 2.2 Blood sample analysis

To obtain serum, blood was allowed to clot in serum gel tubes (IDEXX Laboratories Pty. Ltd., New South Wales, Australia) at room temperature before centrifugation at 4750 *g* for 10 min. Once separated, serum was visually screened for evidence of haemolysis and lipaemia and aliquoted into sterile cryotubes in 0.25 mL increments and frozen at −20°C before being transferred to −80°C within 4 days of collection. Serum was frozen for up to 18 months before being thawed at room temperature and mixed at 2500 rpm for 10 s using a vortexer (Select Bioproducts) prior to analysis. Samples for complete blood count and peripheral blood film analysis and were placed in EDTA tubes (IDEXX Laboratories) and refrigerated at 5°C. All peripheral blood film, complete blood count and serum biochemical analyses were undertaken at IDEXX Laboratories within 5 days of collection using a LaserCyte Dx Haematology Analyser and Catalyst Dx Chemistry Analyser, respectively. Values obtained for haematological and serum biochemistry variables were then compared to previously published reference intervals for bare-nosed wombats (40, 43, 44, 47, 48).

Firstly, having been successfully applied to a broad range of wildlife taxa (23, 49), a multispecies colorimetric peroxidase assay (PHASE™ Haptoglobin Assay, Tridelta, Maynooth, Ireland) was used to measure Haptoglobin (Hp). All assays were performed on the same day using a wet chemistry analyser, the Cobas Integra 400 Plus (Roche Products Pty Ltd., Basel, Switzerland) as per manufacturer instructions. Before analysis, the assay was calibrated with a dedicated calibrator provided with the kit (Haptoglobin Calibrator) followed by a two-level quality control with a dedicated material (Haptoglobin Control). Samples with Hp concentrations above the technical range were diluted 1:5 with phosphate buffered saline diluent (sample/calibrator diluent) and re-analysed as required (19).

Secondly, to obtain a broad overview of AP and other serum protein activity, we analysed serum samples via semiautomated agarose gel electrophoresis (AGE) (Sebia Hydrasys 2 Scan Focusing) with Hydragel 15 Protein(E) alkaline buffered (pH 9.1) agarose gels according to manufacturer’s instructions (Sebia, France). Fraction delimits were automatically determined and electrophoretic curves were examined using Phoresis software (Sebia). Absolute concentrations (g/L) for each protein fraction were calculated by multiplying percentages and total protein concentrations, and albumin:globulin ratios were calculated by dividing albumin by the sum of all globulin fractions (50). Lastly, for evaluation of a point-of-care method for indirect AP protein quantification, blood in sodium citrate tubes was used to conduct micro-erythrocyte sedimentation rate (hereafter ESR) assays within 2 h of collection using routine methods (21). Erythrocyte sedimentation rate assays were performed in duplicate, and the mean value was used for further analyses.

### 2.3 Analytical validation of assays

Analytical validation of assays included assessment of precision via calculation of intra-assay (Hp, AGE, ESR) and inter-assay (AGE) coefficients of variation. For the Hp assay, Hp concentrations were quantified in all samples and two pools of sera were created, one from samples with low and one from samples with high concentrations. Intra-assay coefficients of variation were then determined by analysing 10 consecutive replicates from both pools in a single run. For AGE, intra-assay coefficients of variation were determined by analysing 10 consecutive replicates in the same run using sera from one animal with expected high AP protein concentrations and one animal with expected low concentrations. Inter-assay coefficients of variation were also calculated by performing AGE on four samples over four runs on the same day. Lastly, intra-assay coefficients of variation were determined for ESR by analysing four successive replicates using sera from one animal with sarcoptic mange and one with neurological signs (see below).

We then assessed the accuracy of the Hp assay. Firstly, we conducted linear regression analysis of spike and recovery tests performed in duplicate, diluting a sample from the high concentration pool by 0–100% in 10% increments using sera from the low concentration pool. Secondly, we performed linearity under dilution by diluting a sample from the high concentration pool with phosphate buffered saline diluent (sample/calibrator diluent) in the same % increments.

### 2.4 Clinical validation of assays

Overlap assessments, whereby NSMI changes in association with inflammation are established by the comparison of means between groups with different inflammatory statuses, were conducted to assess the clinical performance of each assay (23). Wombats were assigned to clinical groups depending on clinicopathologic data (51) as follows: (H) apparently healthy wombats having no detectable clinical abnormalities and negative skin scrapes; (M) sarcoptic mange-affected wombats having positive skin scrapes and clinical signs of mange; and, (U) apparently unhealthy wombats having detectable clinical abnormalities but negative skin scrapes and no clinical signs of mange. Group M was further subdivided depending on whether less or greater than 25% of the total body surface was affected by mange (M1 and M2, respectively) (27). Due to sample size limitations, age, sex, season, and sample population were not included in analyses. The Shapiro–Wilk test was then used to determine which NSMI variables were normal (or could be normalised). To assess whether mean NSMI values differed between clinical groups, one-way ANOVAs, or Kruskal-Wallis tests, both with *post hoc* comparisons, were applied to normally distributed and not normally distributed NSMI variables, respectively.

### 2.5 Relationship between sarcoptic mange scores and non-specific markers of inflammation

We also developed a more specific model linking the 14 mange scores associated with an animal to each of its NSMI, in order to better characterise the relationship between apparent disease severity and each NSMI. In the absence of established clinical indicators of mange severity in bare-nosed wombats (i.e., leucograms), we investigated sarcoptic mange scoring as a summary statistic. Specifically, we analysed whether a NSMI could be best predicted based on (i) average mange score, (ii) the highest mange score (i.e., on the 0–10 scale) observed, or (iii) the proportion of body segments with scores >1 (i.e., showing clinical signs of mange). For each individual, we have one or more NSMI and a set of *J* = 14 body-segment scorings of mange ( $x_{i}=\left\{x_{i,1},\ldots x_{i,J}\right\}$ ), where each $x_{i,j}$ is an integer ranging from 0 to 10 indicating mange severity (48). For each NSMI, the expected observed value for animal i , denoted $\mu \left(x_{i}\right)$ , is given by:


$$\mu \left(x_{i}\right)=\alpha_{0}\exp\left(\alpha_{1}\overset{J}{\underset{j=1}{\sum }}{\left(x_{i,j}\right)}^{\alpha_{2}}\right)$$

where $\alpha_{0}$ , $\alpha_{1}$ , and $\alpha_{2}$ , are parameters to be estimated. Variation in the observed NSMI values about the mean were assumed to be consistent with a log-normal distribution. Positive $\alpha_{1}$ indicates the associated NSMI increases with higher mange scores, and *vice-versa*. $\alpha_{2}$ describes how the J mange scores combine to quantify the mange status of an animal. For example, when $\alpha_{2}=1$ the average mange score describes mange status of an animal; however, when $\alpha_{2}>1$ mange status is more influenced by the highest mange score observed on the animal, and when $\alpha_{2}<1$ mange status is more influenced by the number of the 14 mange scores above zero.

For each of the seven NSMI, we fit the model presented above to animals for which we had both an NSMI value and the *J* mange scores. Parameter estimation was performed using Bayesian methods and implemented using the rstan package (see Supplementary materials S1) within the RStudio environment (52). We assumed uninformative priors for all parameters and checked for posterior convergence after a warm-up period of 1,000 iterations (see Supplementary materials S1). Evidence for a relationship between mange and each NSMI was assessed by calculating the credibility that $\alpha_{1}>0$ for 1,000 posterior samples. Group U was not included in this analysis as mange had been excluded as a cause of abnormal health status in these animals.

## 3 Results

Thirty five blood samples were obtained from 33 individual wombats (two animals were sampled on two separate occasions; Supplementary materials S2), and *S. scabiei* were observed in skin scrapes from all wombats visually diagnosed with sarcoptic mange (group M) but no others. For the apparently unhealthy wombats (group U), clinical abnormalities comprised hindlimb lameness (*n* = 1), mandibular abscessation (*n* = 1), lymphocytosis with reactive lymphocytes (*n* = 1), polychromasia and anisocytosis (*n* = 2), and ataxia (*n* = 3). Formalin-fixed tissue samples from 15 sarcoptic mange-affected wombats were subjected to histopathological examination and amyloid material was not observed in any of the viscera evaluated.

### 3.1 Analytical performance of assays

Firstly, the Hp assay was found to be precise: intra-assay coefficients of variation were 3.10 and 8.59% for low and high concentration pools, respectively. Linear regression analyses of spike and recovery and linearity under dilution results showed that the Hp assay was accurate and consistent with a one-to-one relation. For spike and recovery: the 95% confidence intervals for the slope included 1 (0.96–1.07); the y-intercept included 0 (−0.58 to 0.87); and, the *r*^2^-value was 0.92. For linearity under dilution: the 95% confidence intervals for the slope included 1 (0.07–1.37); the y-intercept included 0 (−2.65 to 1.04); and, the *r*^2^-value was 0.88. Recovery percentages ranged from 100 to 150% (mean 129%) for spike and recovery and 100–286% (mean 168%) for linearity under dilution analyses. Haptoglobin was detected in samples from wombats in all four clinical groups (Table 2).

**Table 2:** Sample sizes (*n*) and mean and standard deviation of the mean (SD) values of non-specific markers of inflammation (NSMI), comprising haptoglobin (Hp), albumin (Alb), α-1, α-2, β- and γ-globulins, and erythrocyte sedimentation rate (ESR), for apparently healthy (H), sarcoptic mange-affected (M1 = <25% of the total body surface affected by mange and M2 = >25% of the total body surface affected by mange), and apparently unhealthy (U) bare-nosed wombats (*Vombatus ursinus*) from Tasmania.

NSMI	H			M1 (<25%)			M2 (>25%)			U		
	*n*	Mean	SD	*n*	Mean	SD	*n*	Mean	SD	*n*	Mean	SD
Hp (g/L)	*2*	0.63	0.18	*6*	6.90	9.17	*12*	8.89	7.60	*6*	6.53	2.22
Alb (g/L)	*2*	337.15	30.62	*6*	363.85^a^	22.96	*11*	286.54^b^	64.92	*4*	360.95	26.60
α-1 (g/L)	*2*	31.80	5.94	*6*	26.15	5.42	*11*	35.87	10.32	*4*	30.40	2.12
α-2 (g/L)	*2*	87.50	64.63	*6*	45.70	8.49	*11*	49.98.0	20.19	*4*	46.08	6.04
β (g/L)	*2*	78.90	35.21	*6*	106.75	11.71	*11*	109.65	20.97	*4*	101.13	14.72
γ (g/L)	*2*	54.65^a^	39.95	*6*	109.25^c^	53.53	*11*	161.63^d^	46.25	*4*	98.98^b^	45.68
ESR (mm/h)	*2*	2.75^a^	0.78	*2*	4.90	3.96	*7*	9.26	3.21	*3*	13.16^b^	4.19

Differing superscript characters indicate statistically significant differences between groups, as determined by *post hoc* comparisons.

**Figure 1:**
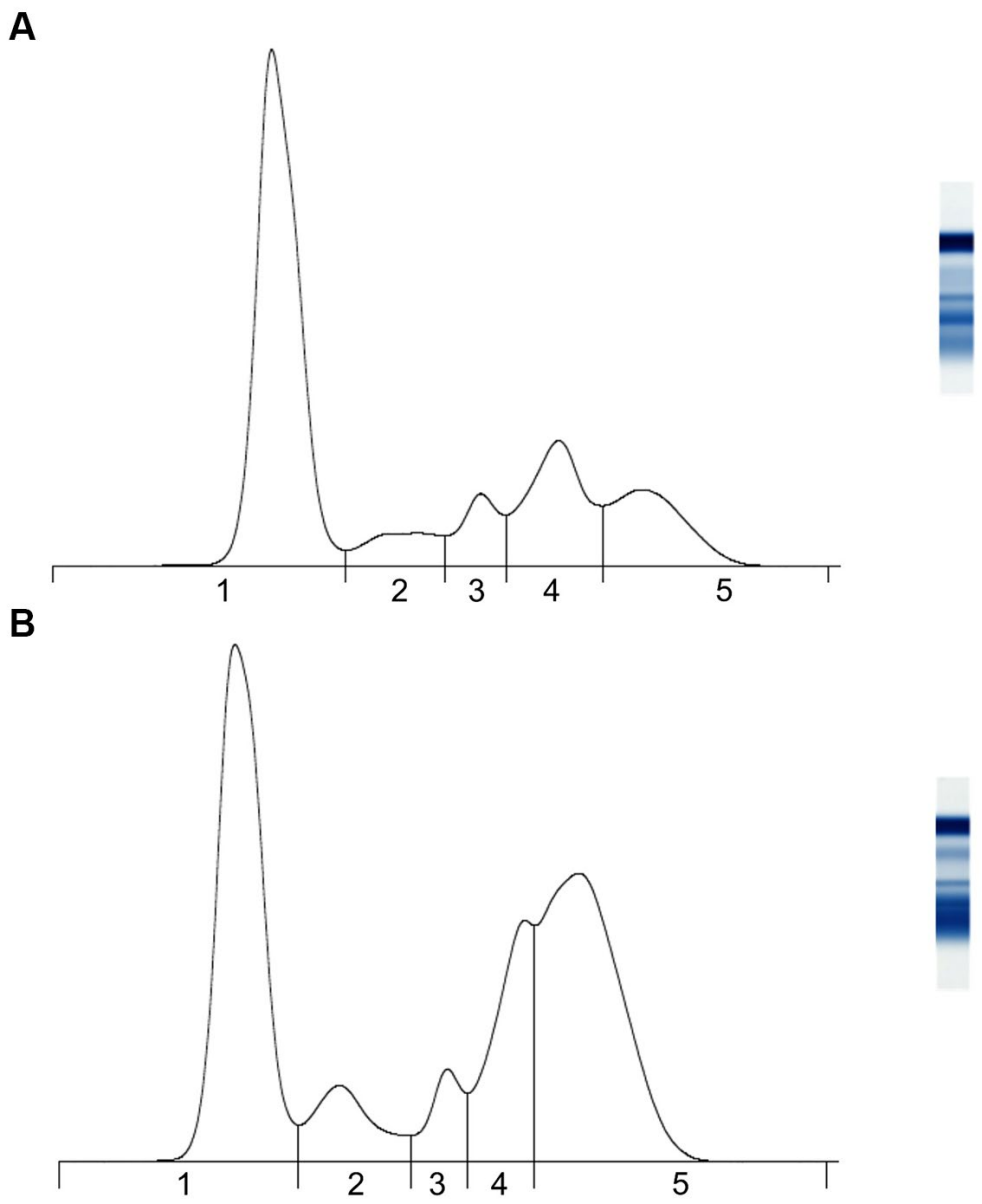
Representative agarose gel protein electrophoretograms of adult bare-nosed wombats (*Vombatus ursinus*) that were **(A)** apparently healthy and **(B)** sarcoptic mange-affected. Vertical lines indicate breaks between (1) albumin, (2) α-1 globulins, (3) α-2 globulins, (4) β-globulins, and (5) γ-globulins.

Secondly, AGE identified five protein fractions comprising albumin, and α-1, α-2, β-, and γ-globulins (Figure 1 and Table 2). Precision was indicated by intra-assay coefficients of variation for each fraction of <0.72, <2.20, <3.10, <1.80, and <1.70%, respectively, and inter-assay coefficients of variation ranges that were 0.38–1.24%, 1.81–3.54%, 0.96–3.02%, 1.38–3.00%, 0.96–4.51%, respectively. Lastly, the precision of the ESR assay was demonstrated by intra-assay coefficients of variation of 3.14% for the mange-affected wombat and 5.18% for the otherwise unhealthy wombat.

### 3.2 Clinical performance of assays

Overlap assessments revealed significant differences in mean serum concentrations among clinical groups for albumin (ANOVA, *F*_3,19_ = 4.07, *p* = 0.022), γ-globulin (Kruskal Wallis, Chi^2^ = 8.82, *p* = 0.033), and ESR (ANOVA, *F*_3,10_ = 5.07, *p* = 0.022) (Table 2). For albumin, the mean concentration of group M2 was lower than all other groups, though the difference was only significant between M2 and M1 (Table 2). There were also non-significant increases in mean concentration from H to M1 and from H to U (Table 2). Statistically significant differences were found between all clinical groups for γ-globulin, with mean concentrations increasing in order from H to U, M1, and M2 (Table 2). For ESR, mean rates increased in order from H to M1, M2, and U, including a significant difference between H and U, and weak evidence of a difference between H and M2 (Table 2). There was no statistical evidence that mean concentrations of Hp, α-1, α-2, or β- globulins differed between any of the groups (Table 2).

### 3.3 Relationship between sarcoptic mange scores and non-specific markers of inflammation

**Figure 2:**
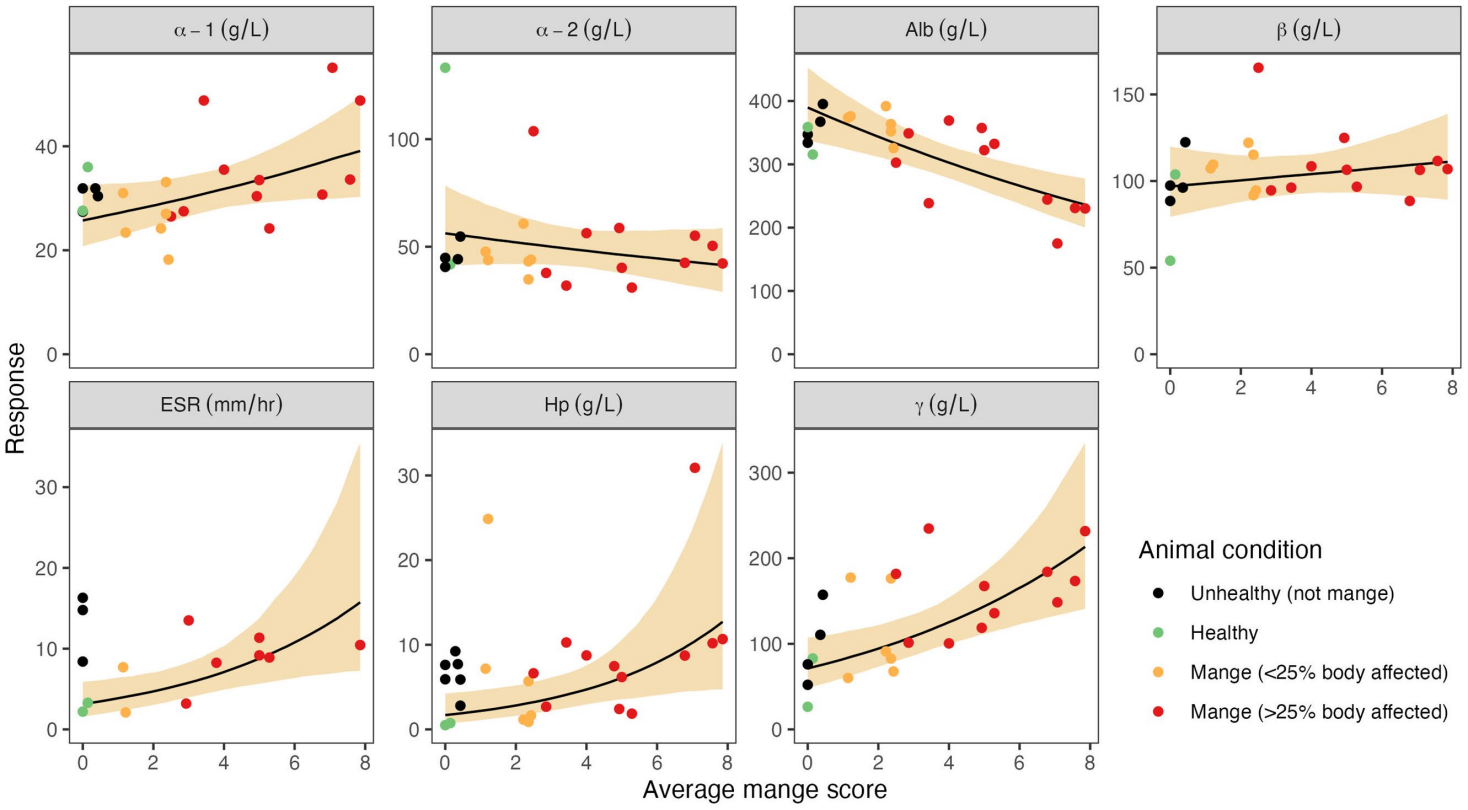
Associations between average mange scores and seven non-specific markers of inflammation (NSMI): haptoglobin (Hp), albumin, α-1, α-2, β, and γ globulins, and erythrocyte sedimentation rate (ESR). Circles depict observed NSMI values from individual wombats, and colours depict the wombat’s clinical group. The solid black line depicts the predicted mean NSMI value when it is assumed to relate to the average mange score (α2 = 1). The shaded region depicts the 95% credible interval associated with the mean. The average mange score is the average of the 14 body-segment mange scores (each an integer in the range 0–10). Note that the data associated with unhealthy animals due to reasons other than mange (black circles), were not used when fitting as the objective of the analysis is to estimate the impact of mange on each NSMI.

Using mange severity as a continuous predictor variable, we found evidence that Hp, γ-globulins, and ESR increased, and albumin decreased, with mange severity, and average mange score was an appropriate measure of mange severity (Table 3 and Figure 2). The relationship between the NSMIs and mange severity was also often best described by $\alpha_{2}<1$ , suggesting that the number of body segments affected by mange-associated skin lesions also provided a useful predictor of an NSMI (Table 3). In concurrence with overlap assessments, there was no evidence that β- globulins were influenced by mange severity (Table 3 and Figure 2). However, according to the modelling approach there was weak evidence for positive and negative relationships between mange severity and α-1 and α-2 globulins, respectively.

**Table 3:** Posterior distribution statistics (mean and 95% credible intervals, CI) for non-specific markers of inflammation (NSMI) in relation to manage severity: serum haptoglobin (Hp), albumin (Alb), α-1, α-2, β-, and γ-globulins, and erythrocyte sedimentation rate (ESR).

NSMI	$\alpha_{0}$		$\alpha_{1}$ *		$\alpha_{2}$ ^		Credibility $\alpha_{1}>0$
	Mean	95% CI	Mean	95% CI	Mean	95% CI	
Hp	1.43	0.46, 4.14	0.050	0.005, 0.126	0.49	0.16, 1.09	**0.987**
Alb	390.41	335.17, 464.24	−0.005	0.019, −0.001	0.97	0.27, 1.46	**0.003**
α-1 globulins	25.89	19.00, 35.28	0.006	−0.005, 0.024	0.60	0.17, 1.40	0.921
α-2 globulins	66.39	47.39, 106.11	−0.019	−0.053, 0.000	0.30	0.14, 1.39	0.024
β-globulins	93.95	74.63, 117.34	0.004	−0.006, 0.021	0.38	0.15, 1.09	0.818
γ-globulins	58.36	34.75, 95.78	0.031	0.007, 0.076	0.44	0.15, 1.23	**1.000**
ESR	3.14	1.52, 5.86	0.030	0.002, 0.081	0.52	0.17, 1.18	**0.982**

Units are depicted in Figure 2. Credibility column describes evidence that mange has a positive relation with the NSMI; values greater than 0.975 and less than 0.025 suggest evidence for a positive and negative relation, respectively (bold).

*Positive $\alpha_{1}$ indicates NSMI increases with higher mange scores, negative $\alpha_{1}$ indicates NSMI decreases with higher mange scores.

^ $\alpha_{2}=1$ indicates that average mange score provides the best description of mange severity, $\alpha_{2}<1$ indicates that mange severity is best described by the number of body segments affected by mange-associated skin lesions.

## 4 Discussion

Sarcoptic mange threatens the health, welfare, and, in some cases, conservation status of a growing number of mammalian wildlife species worldwide (3). This study addresses knowledge gaps associated with this panzootic disease by developing tools for measuring the host immune response that could inform management interventions and investigating contributions of the AP response to mange pathophysiology (51). Here, we present clinical and analytical validation of Hp, AGE, and ESR assays for novel use in wombats. We provide evidence that NSMI representing AP and other serum proteins undergo changes in association with sarcoptic mange in this species, but that systemic amyloidosis is not a definitive corollary of the AP response.

Acute phase responses have been documented in association with sarcoptic mange in a small number of wild animal species (1, 7, 9, 16, 28). Haptoglobin is a frequently assessed NSMI and in most species has been found to be a positive AP protein in association with mange presence or severity (7, 9, 16, 28). Consistent with other literature, we found evidence that Hp concentrations correlated positively with mange scores in wombats. Mean Hp for healthy wombats (group H) was consistent with other species (53), and increased by 10–14 fold for the diseased groups, suggesting that Hp may be a moderate to major AP protein in wombats (54). That inter-group differences did not differ statistically, as has been the case for most studies using this assay (23), suggests that the standard overlap performance approach is not very powerful. Variation in AP protein concentrations between individual animals is common even within single disease contexts (30, 55), including for Hp (32), and in association with mange (9, 16). Our observations of marked intra-group variation (i.e., some animals in M1 had relatively high Hp concentrations and vice versa for some in M2) most-likely reflected differences in the degree of immune activation, for example unexpectedly high concentrations may have been related to the presence of secondary bacterial infections (27), or other comorbidities (15), while stressors (17) or immune exhaustion may have contributed to lower than expected concentrations. As Hp is also thought to be associated with chronic inflammation in some species (53), the timepoint post-infection may be a particularly important consideration and longitudinal studies would be beneficial (14, 20).

In the study of sarcoptic mange in free-living wildlife, serum protein electrophoresis has seen fewer applications (29, 48, 56, 57) than direct assays for specific AP proteins such as Hp, serum amyloid A, α-1-acid glycoprotein, ceruloplasmin, and acetylcholinesterase (1, 7, 9, 16, 28). One reason may be that the usefulness of serum protein electrophoresis in certain wildlife species (e.g., ungulates and rodents) is superseded by the availability of commercial immunoassays for specific AP proteins that were developed for use in closely related domestic animals and for which antibody cross-reactivity has been demonstrated (1, 7). However, since the bare-nosed wombat is genetically distinct from domestic taxa, it was unknown which AP proteins would be optimal to target (9, 23, 31, 58). Indeed, the lack of cross-reactivity found by a pilot study, which used serum obtained from zoo-housed bare-nosed wombats from the United States of America and commercial immunoassays for serum amyloid A (i.e., LZ- SAA Assay and VET-SAA Assay, Eiken Chemical Co., Japan, and Phase™ Range Multispecies SAA ELISA kit, Tridelta, Ireland) and C-reactive protein (Canine CRP Assay, Randox Laboratories, United Kingdom), suggested that species-specific assays would need to be developed (Supplementary materials S3). Serum protein electrophoresis therefore represented an effective means of providing an overview of serum protein activity for a species in which the AP response has not previously been investigated, with a view to directing future research towards the most promising candidate proteins. While cellulose acetate electrophoresis, a now obsolete method of serum protein electrophoresis, has been applied previously in this host-pathogen system (29), the present study expanded upon this analysis by: increasing sample size; using an agarose gel method; performing analytical validation; accounting for differences in disease severity; and, identifying specific AP protein targets for future research.

Serum protein electrophoresis permits valid albumin quantification relative to colorimetric methods (59), and we found strong overlap and model-supported evidence that albumin is a negative AP protein in wombats (i.e., it decreases in concentration in association with disease, as evidenced by groups U and M). Within the mange context, this finding is consistent with colorimetric albumin quantitation in capybara (16), and previous findings in bare-nosed wombats based on cellulose acetate electrophoresis (29), but data using methods comparable to ours (i.e., AGE) are lacking for additional wildlife hosts. A second advantage of serum protein electrophoresis is that, in place of more complicated but less coarse methods, such as serum proteome profiling (60), it can reveal α- and β-globulin fractions (61) worth targeting with more specific assays. For these fractions, an AP response characteristically manifests as increased α-1, α-2, and β-2, and decreased β-1, relative to healthy controls (62).

Though differences between clinical groups were not detected by overlap assessment, our continuous model found evidence that mange severity is positively associated with increasing α-1 globulins in wombats. In other mammalian species, increased α-1 globulin concentrations have been associated with chronic disease (55). However, within the sarcoptic mange context, previous investigations that relied on overlap assessments in wombats and other species did not reveal differences between healthy and mange-affected groups (29, 57). Nonetheless, our model findings suggest that proteins known to migrate to the α-1 fraction (e.g., α-1-antitrypsin, α-1-acid glycoprotein, α-1-antichymotrypsin, α-1-fetoprotein, and α-1-lipoprotein) may play a role in the host response to sarcoptic mange (31). Although the precise components of the α-1 fraction are unknown in wombats, the AP response is thought to be highly conserved across species (21). As α-1-acid glycoprotein, in particular, has been found to be a major and moderate AP protein in mange-affected Alpine ibex (9) and capybara (16), respectively, it represents a promising avenue for investigation in wombats.

In contrast to generalisations regarding the characteristic AP response to inflammation (62), we found that α-2 globulins had a negative relationship to sarcoptic mange severity. Though surprising, particularly as Hp is typically a major component of the α-2 globulin fraction (31), negative relationships between this fraction and sarcoptic mange have also been reported in Pyrenean chamois (*Rupicapra pyrenaica*), Iberian ibex (57), and coyotes (*Canis latrans*) (56). Such findings may be a function of variation in disease duration, since, aside from Hp (22), α-2 globulins are thought to signal acute inflammation (63), whereas mange is a disease of chronicity (64). Additionally, it could indicate that unknown negative AP proteins migrate to the α-2 fraction (55) in association with mange.

That β-globulins did not appear to be influenced by mange severity may be due to the lack of resolution between β-1 and β-2 fractions in our electrophoretograms. This may have led to increases in positive AP proteins within the β-2 fraction [e.g., C-reactive protein (65)] being masked by decreases in negative AP proteins in β-1 [e.g., transferrin (31)]. Pursuit of serum protein electrophoresis methods capable of resolving β-1 and β-2 fractions, alongside validation of other specific AP protein assays that target α-2, β-1, and β-2 globulins, such as ceruloplasmin, C-reactive protein, and transferrin, respectively, is likely to be of value. In particular, ceruloplasmin has already been shown to be a positive AP protein in association with mange in Alpine ibex (9) and dromedary camels (28), and the assay’s functional nature mean it could be readily applied to wombat samples (9).

Though not strictly part of the AP response, serum protein electrophoresis enables assessment of immunoglobulin activity via the γ-globulin fraction without needing to validate species-specific immunoassays. Increased γ-globulins are suggestive of an immunoglobulin response (55) and associated with chronic immune stimulation (14, 20). We found increases in mean γ-globulin concentrations in diseased groups relative to healthy animals, indicating immunoglobulin production in association with sarcoptic mange. The relative increases observed in groups M1 and M2 are consistent with findings from healthy versus mange-affected coyotes (56), and support evidence that γ-globulin concentrations increased with mange severity. Though our overlap assessments contrast with previous reports from bare-nosed wombats (29), chamois, and ibex (57), the stimulation of immunoglobulin-G production in mange-affected hosts has been demonstrated in the latter two species (10, 57), as well as red fox (*Vulpes vulpes*) (66) and European rabbit (*Oryctolagus cuniculus*) (67). An immunoglobulin response has not previously been documented in mange-affected wombats, but due to the non-specific nature of AGE it remains unknown whether this finding was associated with production of immunoglobulins specific to *S. scabiei*, or other simultaneous pathological processes (e.g., secondary bacterial infection) (68). Given the role of immunoglobulin responses in the development of immunity and as tools for enhancing mange diagnosis and surveillance, the utility of species-specific enzyme-linked immunosorbent assays could be investigated to further elucidate the humoral response to sarcoptic mange in wombats (10, 57, 69).

While the above methods of AP response assessment require specialised equipment and personnel, ESR is a low-cost, low-tech alternative that can be performed in the field with rapid results (33, 70). Though this type of assay has never been used in association with sarcoptic mange, its practicality as a tool to inform management decisions has been demonstrated by increases in ESR correlating with other known measures of inflammation and in association with diseased states (21, 33, 70). Erythrocyte sedimentation rate assays have also been shown analytically to perform as well as commercially available kits, such as for the Westergren method (21, 70). In agreement with these studies, our preliminary assessment of this assay in wombats found that it had good analytical and clinical performance. Reflecting activation of a systemic immune response and the presence of inflammatory serum proteins causing red blood cells to aggregate and precipitate at a faster rate (70), diseased wombats had increased ESRs relative to group H, and we found evidence that ESR increased in conjunction with mange severity. Though sample sizes were smaller than for Hp and AGE, our findings suggest that this assay could be a useful means of assessing systemic health and supporting management decisions in wombats, particularly in the field and other resource-limited or time-critical settings.

Amyloidosis is a known mechanism of mange pathophysiology and the accumulation of amyloid material in the tissues of mange-affected Iberian ibex has been shown to correlate with AP protein concentrations (1). Contrastingly, and supported by previous research (29), our findings indicate that amyloidosis is not part of mange pathogenesis in wombats and suggest that the AP response in this species is mostly comprised of proteins that contribute less significantly to immunopathology (e.g., Hp) than others (20). As AP protein expression can vary among species and disease processes (71), it is possible that the absence of systemic amyloidosis in the wombats examined in this study represents yet another species-specific difference in sarcoptic mange immunopathology (4). Nonetheless, investigations into the activity of specific AP proteins that contribute to systemic amyloidosis would help to understand the mechanism and relevance of this finding. For example, serum amyloid A has been linked to systemic amyloidosis in numerous species (13, 31, 72) and in association with sarcoptic mange (1). Though development of a species-specific assay would likely be required, knowledge of the role of this AP protein in wombats would likely be of value.

Advancing abilities to assess sarcoptic mange severity is valuable for informing management decisions, particularly because commonly used clinicopathological indicators such as complete blood count and serum biochemistry variables (excluding serum protein electrophoretic fractions) have not yielded consistent results among studies in wombats (44). We therefore used sarcoptic mange scoring, which is widely used in wombats (40, 41) and other species, to assess the clinical performance of NSMI assays. This method has been validated as a diagnostic tool for mange in wombats (45), but there is limited understanding of the extent to which scores accurately reflect an animal’s systemic disease state. Our analyses suggest that the scoring approach, and to a lesser degree the overall number of body segments affected by mange-associated skin lesions, is a reasonable proxy measure of the AP response. There are, therefore, benefits of using this method over AP assays, such as that it is non-invasive and requires minimal equipment and resources. However, the individual variation observed in this study for all NSMIs investigated also shows that the severity of visual clinical signs may not always align with systemic disease impacts, which in some cases may be underrepresented. Further research is therefore needed to disentangle disparities between observed disease severity and AP response, for example through assessment of correlations among assays and mange scores over time.

As for most wildlife studies (23), capacity to conduct complete analytical validation was limited by difficulties in obtaining sufficiently large sample sizes and the prohibitive costs of assay kits and equipment (excluding the ESR assay). Acknowledging our small sample size the extent of assay validation performed here in terms of precision/accuracy assessment is comparable to other examples in the literature (21, 23, 59). We found the Hp assay to be precise and accurate with good linearity and acceptable recovery performance, relative to recommendations (23), and the precision of the AGE and ESR was also comparable to other species (21, 61). Establishing the clinical validity of NSMI assays in novel wildlife species is similarly challenging, particularly in free-living animals with naturally acquired disease. Though clinical assay validation may be performed in captive wild animals with experimentally-induced inflammation under controlled laboratory conditions (73), ethical and practical considerations, including wombat availability in captivity, meant that this was not feasible for the present study. The findings presented here therefore emphasise the limitations of overlap performance testing, the recommended method of clinical validation (23), in ecological systems, where: sample sizes can be limited; natural variation is commonplace (25); knowledge about factors such as duration of disease or co-infections is lacking (23, 54, 55); and, the true health status of control animals is unknown or biassed (31).

For example, overlap assessments in the present study may have been affected by the small sample size, particularly for group H, and impact of variable sample processing times (1–5 days) on complete blood counts, which dictated (in part) animal allocation to groups H or U (74). Also, owing to the challenge of obtaining sufficiently large samples of animals with well-described health information (23, 54), use of groups based on coarse assessments of disease status is often necessary for clinical validation studies in captive and free-living wildlife, such as presence/absence of cryptosporidiosis in eastern indigo snakes (*Drymarchon couperi*) (70), and healthy/diseased southern white rhinoceros (*Ceratotherium simum simum*) (14). While the severity of mange-related inflammation was likely adequately described by groups M1 and M2 (27), clinicopathological data was insufficient to determine aetiology or chronicity for pathologies represented in group U (see Results). Therefore, this group represented a spectrum of inflammatory severity and chronicity that was unaccounted for and may have influenced findings. If possible, future studies validating NSMI assays in wombats would benefit from basing clinical groups for overlap assessments on differing inflammation intensity, or through longitudinal study. Additional limitations of this study include that our sample size did not permit investigation of non-disease effects on NSMI, nor assessment of correlation among assays. As the influence of non-disease factors such as sex and season on the AP response has been demonstrated in other wildlife species (14, 32, 55), including mange-associated variation between sample populations (57), further investigation in wombats is warranted.

This work provides evidence linking the immune response and sarcoptic mange severity in bare-nosed wombats. We demonstrate the potential utility of Hp, AGE, and ESR assays, and identify candidate AP proteins that could further contribute to a panel of health-monitoring and decision-making tools for this species (32). Through the future establishment of reference intervals, calculation of sensitivity and specificity (17), and longitudinal studies that investigate changes in the immune response over time, these assays have scope to: inform management practices that improve health and welfare outcomes for mange-affected animals (7); investigate population-level differences in baseline AP protein levels (57), with a view to identifying those potentially more at risk of severe impacts and thereby direct mitigation strategies (7); and, be applied to a range of other clinical and population health scenarios *in* and *ex situ*, such as toxoplasmosis (75, 76). Our insights also provide further evidence of the importance and usefulness of NSMI investigations in the sarcoptic mange-context (1, 7, 9, 16), with particular relevance to additional Australian species impacted by sarcoptic mange, including the koala (77).

## Data availability statement

The original contributions presented in the study are included in the article/Supplementary material, further inquiries can be directed to the corresponding author.

## Ethics statement

The animal study was approved by the University of Tasmania Animal Ethics Committee. The study was conducted in accordance with the local legislation and institutional requirements.

## Author contributions

VW: Writing – review & editing, Writing – original draft, Validation, Project administration, Methodology, Investigation, Funding acquisition, Formal analysis, Data curation, Conceptualization. SR: Funding acquisition, Writing – review & editing, Supervision, Methodology, Formal analysis. SC: Writing – review & editing, Supervision, Methodology, Funding acquisition, Conceptualization. CN-N: Funding acquisition, Writing – review & editing. CC: Writing – review & editing, Methodology. GR: Writing – review & editing, Supervision, Methodology, Formal analysis.

## References

[1] Espinosa J Ráez-Bravo A López-Olvera JR Pérez JM Lavín S Tvarijonaviciute A. Histopathology, microbiology and the inflammatory process associated with *Sarcoptes scabiei* infection in the Iberian ibex, *Capra pyrenaica*. Parasit Vectors. (2017) 10:596. doi: 10.1186/s13071-017-2542-5, PMID: 29202802 PMC5715492

[2] Turchetto S Obber F Rossi L D'Amelio S Cavallero S Poli A. Sarcoptic mange in wild Caprinae of the Alps: could pathology help in filling the gaps in knowledge? Front Vet Sci. (2020) 7:193. doi: 10.3389/fvets.2020.00193, PMID: 32432130 PMC7214924

[3] Escobar LE Carver S Cross PC Rossi L Almberg ES Yabsley MJ. Sarcoptic mange: an emerging panzootic in wildlife. Transbound Emerg Dis. (2022) 69:927–42. doi: 10.1111/tbed.14082, PMID: 33756055

[4] Naesborg-Nielsen C Wilkinson V Mejia-Pacheco N Carver S. Evidence underscoring immunological and clinical pathological changes associated with *Sarcoptes scabiei* infection: synthesis and meta-analysis. BMC Infect Dis. (2022) 22:658. doi: 10.1186/s12879-022-07635-5, PMID: 35902827 PMC9335973

[5] Pence DB Ueckermann E. Sarcoptic mange in wildlife. Rev Sci Tech. (2002) 21:385–98. doi: 10.20506/rst.21.2.1335 11974622

[6] Oleaga A Casais R Prieto JM Gortázar C Balseiro A. Comparative pathological and immunohistochemical features of sarcoptic mange in five sympatric wildlife species in northern Spain. Eur J Wildl Res. (2012) 58:997–1000. doi: 10.1007/s10344-012-0662-y

[7] Ráez-Bravo A Granados JE Ceron JJ Cano-Manuel FJ Fandos P Pérez JM. Acute phase proteins increase with sarcoptic mange status and severity in Iberian ibex (*Capra pyrenaica*, Schinz 1838). Parasitol Res. (2015) 114:4005–10. doi: 10.1007/s00436-015-4628-3, PMID: 26227139

[8] Meza Cerda MI Gray R Thomson PC Butcher L Simpson K Cameron A. Developing immune profiles of endangered Australian sea lion (*Neophoca cinerea*) pups within the context of endemic hookworm (*Uncinaria sanguinis*) infection. Front Vet Sci. (2022) 9:824584. doi: 10.3389/fvets.2022.824584, PMID: 35529837 PMC9069138

[9] Rahman MM Lecchi C Fraquelli C Sartorelli P Ceciliani F. Acute phase protein response in alpine ibex with sarcoptic mange. Vet Parasitol. (2010) 168:293–8. doi: 10.1016/j.vetpar.2009.12.001, PMID: 20036058

[10] Ráez-Bravo A Granados JE Serrano E Dellamaria D Casais R Rossi L. Evaluation of three enzyme-linked immunosorbent assays for sarcoptic mange diagnosis and assessment in the Iberian ibex, *Capra pyrenaica*. Parasit Vectors. (2016) 9:558. doi: 10.1186/s13071-016-1843-4, PMID: 27769278 PMC5073795

[11] Martínez IZ Oleaga Á Sojo I García-Iglesias MJ Pérez-Martínez C García Marín JF. Immunohistochemical assessment of immune response in the dermis of *Sarcoptes scabiei*—infested wild carnivores (wolf and fox) and ruminants (chamois and red deer). Animals. (2020) 10:1146. doi: 10.3390/ani10071146, PMID: 32640758 PMC7401513

[12] Sarasa M Rambozzi L Rossi L Meneguz PG Serrano E Granados JE. *Sarcoptes scabiei*: specific immune response to sarcoptic mange in the Iberian ibex *Capra pyrenaica* depends on previous exposure and sex. Exp Parasitol. (2010) 124:265–71. doi: 10.1016/j.exppara.2009.10.008, PMID: 19857492

[13] Cray C. Acute phase proteins in animals In: Conn PM, editor. Progress in molecular biology and translational science, vol. 105. London: Academic Press (2012). 113–50. 10.1016/B978-0-12-394596-9.00005-6 PMC7149966 22137431

[14] Petersen HH Stenbak R Blaabjerg C Krogh AKH Bertelsen MF Buss P. Development of a quantitative immunoassay for serum haptoglobin as a putative disease marker in the southern white rhinoceros (*Ceratotherium simum simum*). J Zoo Wildl Med. (2022) 53:141–52. doi: 10.1638/2020-0010, PMID: 35339159

[15] Thurber MI Singleton C Cray C. Reference intervals for acute phase proteins for koalas (*Phascolarctos cinereus*) at the San Diego zoo. J Zoo Wildl Med. (2019) 50:735–8. doi: 10.1638/2018-0227, PMID: 33517648

[16] Bernal L Feser M Martinez-Subiela S Garcia-Martinez JD Ceron JJ Tecles F. Acute phase protein response in the capybara (*Hydrochoerus hydrochaeris*). J Wildl Dis. (2011) 47:829–35. doi: 10.7589/0090-3558-47.4.829 22102653

[17] Stanton JJ Cray C Rodriguez M Arheart KL Ling PD Herron A. Acute phase protein expression during elephant endotheliotropic herpesvirus-1 viremia in Asian elephants (*Elephas maximus*). J Zoo Wildl Med. (2013) 44:605–12. doi: 10.1638/2012-0174R1.1, PMID: 24063088

[18] Gelain ME Bonsembiante F. Acute phase proteins in marine mammals: state of art, perspectives and challenges. Front Immunol. (2019) 10:1220. doi: 10.3389/fimmu.2019.01220, PMID: 31191557 PMC6549532

[19] Edwards KL Miller MA Siegal-Willott J Brown JL. Serum health biomarkers in African and Asian elephants: value ranges and clinical values indicative of the immune response. Animals. (2020) 10:1756. doi: 10.3390/ani10101756, PMID: 32992555 PMC7601509

[20] Glidden CK Beechler B Buss PE Charleston B de Klerk-Lorist LM Maree FF. Detection of pathogen exposure in African buffalo using non-specific markers of inflammation. Front Immunol. (2017) 8:1944. doi: 10.3389/fimmu.2017.01944 29375568 PMC5768611

[21] Adamovicz L Baker SJ Kessler E Kelly M Johnson S Winter J. Erythrocyte sedimentation rate and hemoglobin-binding protein in free-living box turtles (*Terrapene* spp.). PLoS One. (2020) 15:e0234805. doi: 10.1371/journal.pone.0234805, PMID: 32555669 PMC7299368

[22] Cray C Arheart KL Hunt M Clauss T Leppert LL Roberts K. Acute phase protein quantitation in serum samples from healthy Atlantic bottlenose dolphins (*Tursiops truncatus*). J Vet Diagn Invest. (2013) 25:107–11. doi: 10.1177/1040638712467986, PMID: 23242666

[23] Hooijberg EH Cray C. Acute phase reactants in nondomesticated mammals - a veterinary clinical pathology perspective. Vet Clin Pathol. (2023) 52:19–36. doi: 10.1111/vcp.13189 36289012

[24] Bondo KJ Macbeth B Schwantje H Orsel K Culling D Culling B. Health survey of boreal caribou (*Rangifer tarandus caribou*) in northeastern British Columbia, Canada. J Wildl Dis. (2019) 55:544–62. doi: 10.7589/2018-01-018, PMID: 30605390

[25] Busch JD Van Andel R Cordova J Colman RE Keim P Rocke TE. Population differences in host immune factors may influence survival of Gunnison's prairie dogs (*Cynomys gunnisoni*) during plague outbreaks. J Wildl Dis. (2011) 47:968–73. doi: 10.7589/0090-3558-47.4.968, PMID: 22102668

[26] Fritze M Puechmaille SJ Costantini D Fickel J Voigt CC Czirjak GA. Determinants of defence strategies of a hibernating European bat species towards the fungal pathogen *Pseudogymnoascus destructans*. Dev Comp Immunol. (2021) 119:104017. doi: 10.1016/j.dci.2021.104017, PMID: 33476670

[27] Dinler Ay C Tuna GE Ekren Asici GS Ulutas B. Effects of the clinical severity of disease and concomitant pyoderma on serum acute-phase proteins concentrations in dogs with sarcoptic mange. Vet Dermatol. (2022) 33:378–83. doi: 10.1111/vde.13095, PMID: 35670652

[28] Hassan H Kamr A Arbaga AA. Acute phase proteins, trace elements and cytokines expression as a diagnostic and prognostic biomarker in diseased camel. J Curr Vet Res. (2020) 2:93–100. doi: 10.21608/jcvr.2020.90229

[29] Skerratt LF Middleton D Beveridge I. Distribution of life cycle stages of *Sarcoptes scabiei* var. *wombati* and effects of severe mange on common wombats in Victoria. J Wildl Dis. (1999) 35:633–46. doi: 10.7589/0090-3558-35.4.633, PMID: 10574522

[30] Coon CA Warne RW Martin LB. Acute-phase responses vary with pathogen identity in house sparrows (*Passer domesticus*). Am J Physiol Regul Integr Comp Physiol. (2011) 300:R1418–25. doi: 10.1152/ajpregu.00187.2010, PMID: 21346241

[31] Depauw S Delanghe J Whitehouse-Tedd K Kjelgaard-Hansen M Christensen M Hesta M. Serum protein capillary electrophoresis and measurement of acute phase proteins in a captive cheetah (*Acinonyx jubatus*) population. J Zoo Wildl Med. (2014) 45:497–506. doi: 10.1638/2013-0111R1.1, PMID: 25314816

[32] Lee KA Goetting VS Tell LA. Inflammatory markers associated with trauma and infection in red-tailed hawks (*Buteo jamaicensis*) in the USA. J Wildl Dis. (2015) 51:860–7. doi: 10.7589/2014-04-093, PMID: 26280876

[33] Yarborough E Glidden C Coon C Couch C Sisson D Johns J. Exploring the use of erythrocyte sedimentation rate as an inflammatory marker for free-ranging wildlife: a case study in African buffalo (*Syncerus caffer*). J Wildl Dis. (2022) 58:298–308. doi: 10.7589/JWD-D-21-00114, PMID: 35276000

[34] Fraser TA Holme R Martin A Whiteley P Montarello M Raw C. Expanded molecular typing of *Sarcoptes scabiei* provides further evidence of disease spillover events in the epidemiology of sarcoptic mange in Australian marsupials. J Wildl Dis. (2019) 55:231–7. doi: 10.7589/2018-04-101, PMID: 30096035

[35] Simpson K Johnson CN Carver S. *Sarcoptes scabiei*: the mange mite with mighty effects on the common wombat (*Vombatus ursinus*). PLoS One. (2016) 11:e0149749. doi: 10.1371/journal.pone.0149749, PMID: 26943790 PMC4778766

[36] Ferreyra HDV Rudd J Foley J Vanstreels RET Martin AM Donadio E. Sarcoptic mange outbreak decimates south American wild camelid populations in san Guillermo National Park, Argentina. PLoS One. (2022) 17:e0256616. doi: 10.1371/journal.pone.0256616, PMID: 35061672 PMC8782313

[37] Martin AM Burridge CP Ingram J Fraser TA Carver S. Invasive pathogen drives host population collapse: effects of a travelling wave of sarcoptic mange on bare-nosed wombats. J Appl Ecol. (2018) 55:331–41. doi: 10.1111/1365-2664.12968

[38] Tamura J Ingram J Martin AM Burridge CP Carver S. Contrasting population manipulations reveal resource competition between two large marsupials: bare-nosed wombats and eastern grey kangaroos. Oecologia. (2021) 197:313–25. doi: 10.1007/s00442-021-04959-y, PMID: 34095983

[39] Carver S Lewin ZM Burgess LG Wilkinson V Whitehead J Driessen MM. Density independent decline from an environmentally transmitted parasite. Biol Lett. (2023) 19:20230169. doi: 10.1098/rsbl.2023.0169, PMID: 37607579 PMC10444343

[40] Wilkinson V Takano K Nichols D Martin A Holme R Phalen D. Fluralaner as a novel treatment for sarcoptic mange in the bare-nosed wombat (*Vombatus ursinus*): safety, pharmacokinetics, efficacy and practicable use. Parasit Vectors. (2021) 14:1–21. doi: 10.1186/s13071-020-04500-9 33407820 PMC7789169

[41] Martin AM Richards SA Fraser TA Polkinghorne A Burridge CP Carver S. Population-scale treatment informs solutions for control of environmentally transmitted wildlife disease. J Appl Ecol. (2019) 56:2363–75. doi: 10.1111/1365-2664.13467

[42] Old JM Skelton CJA Stannard HJ. The use of Cydectin® by wildlife carers to treat sarcoptic mange in free-ranging bare-nosed wombats (*Vombatus ursinus*). Parasitol Res. (2021) 120:1077–90. doi: 10.1007/s00436-020-07012-8, PMID: 33438043

[43] Skerratt LF. Clinical response of captive common wombats (*Vombatus ursinus*) infected with *Sarcoptes scabiei* var. *wombati*. J Wildl Dis. (2003) 39:179–92. doi: 10.7589/0090-3558-39.1.179, PMID: 12685082

[44] Hartley M English A. *Sarcoptes scabei* var. *wombati* infection in the common wombat (*Vombatus ursinus*). Eur J Wildl Res. (2005) 51:117–21. doi: 10.1007/s10344-005-0080-5

[45] Fraser TA Martin A Polkinghorne A Carver S. Comparative diagnostics reveals PCR assays on skin scrapings is the most reliable method to detect *Sarcoptes scabiei* infestations. Vet Parasitol. (2018) 251:119–24. doi: 10.1016/j.vetpar.2018.01.007, PMID: 29426467

[46] Martin A Skerratt L Carver S. Sarcoptic mange in Australian wildlife. Fact Sheet Wildlife Health Australia. (2017) 1:1–11.

[47] Booth R. Wombats: care and treatment of sick, injured and orphaned animals In: Wildlife in Australia: healthcare and management. ed. Dryden DI Sydney: University of Sydney (1999)

[48] Ruykys L Breed B Schultz D Taggart D. Effects and treatment of sarcoptic mange in southern hairy-nosed wombats (*Lasiorhinus latifrons*). J Wildl Dis. (2013) 49:312–20. doi: 10.7589/2012-10-256, PMID: 23568906

[49] Bertelsen MF Kjelgaard-Hansen M Grondahl C Heegaard PM Jacobsen S. Identification of acute phase proteins and assays applicable in nondomesticated mammals. J Zoo Wildl Med. (2009) 40:199–203. doi: 10.1638/2007-0125.1, PMID: 19368263

[50] Rosenberg JF Hernandez JA Wellehan James FX Crevasse SE Cray C Stacy NI. Diagnostic performance of inflammatory markers in gopher tortoises (*Gopherus polyphemus*). J Zoo Wildl Med. (2018) 49:765–9. doi: 10.1638/2017-0211.1, PMID: 30212346

[51] Bossart GD Romano TA Peden-Adams MM Schaefer AM Rice CD Fair PA. Comparative innate and adaptive immune responses in Atlantic bottlenose dolphins (*Tursiops truncatus*) with viral, bacterial, and fungal infections. Front Immunol. (2019) 10:1125. doi: 10.3389/fimmu.2019.01125, PMID: 31231361 PMC6558379

[52] RStudio Team. RStudio: integrated development for R, vol. 42. Boston, MA: RStudio, Inc (2015). 14 p.

[53] Isaza R Wiedner E Hiser S Cray C. Reference intervals for acute phase protein and serum protein electrophoresis values in captive Asian elephants (*Elephas maximus*). J Vet Diagn Invest. (2014) 26:616–21. doi: 10.1177/1040638714543923, PMID: 25057161

[54] Tobin K Zimmerman D Rasmussen J Hilton CD Junge RE Armstrong D. Establishment of acute-phase and serum protein electrophoresis preliminary reference values for pronghorn (*Antilocapra americana*). J Zoo Wildl Med. (2020) 51:321–5. doi: 10.1638/2018-0226, PMID: 32549561

[55] Miller S Cray C Schaefer AM Reif JS Roberts K Bossart GD. Assessment of serum amyloid a, haptoglobin, and protein electrophoresis in clinically healthy and abnormal bottlenose dolphins (*Tursiops truncatus*). Aquat Mamm. (2020) 46:131–6. doi: 10.1578/AM.46.2.2020.131

[56] Pence DB Windberg LA Pence BC Sprowls R. The epizootiology and pathology of sarcoptic mange in coyotes, *Canis latrans*, from South Texas. J Parasitol. (1983) 69:1100–15. doi: 10.2307/3280873, PMID: 6425486

[57] Lastras ME Pastor J Marco I Ruiz M Viñas L Lavín S. Effects of sarcoptic mange on serum proteins and immunoglobulin G levels in chamois (*Rupicapra pyrenaica*) and Spanish ibex (*Capra pyrenaica*). Vet Parasitol. (2000) 88:313–9. doi: 10.1016/S0304-4017(99)00221-6, PMID: 10714470

[58] Tothova C Nagy O Kovac G. Serum proteins and their diagnostic utility in veterinary medicine: a review. Vet Med. (2016) 61:475–96. doi: 10.17221/19/2016-VETMED

[59] Cray C. Protein electrophoresis of non-traditional species: a review. Vet Clin Pathol. (2021) 50:478–94. doi: 10.1111/vcp.13067, PMID: 34881455

[60] Chow BA Donahue SW Vaughan MR McConkey B Vijayan MM. Serum immune-related proteins are differentially expressed during hibernation in the American black bear. PLoS One. (2013) 8:e66119. doi: 10.1371/journal.pone.0066119, PMID: 23825529 PMC3692520

[61] Meyer A Emerson JA Rainwater KL Haefele H Arheart KL Hammond E. Assessment of capillary zone electrophoresis and serum amyloid a quantitation in clinically normal and abnormal southern white rhinoceros (*Ceratotherium simum simum*) and southern black rhinoceros (*Diceros bicornis minor*). J Zoo Wildl Med. (2022) 53:319–30. doi: 10.1638/2021-0072, PMID: 35758573

[62] Gray R Canfield P Rogers T. Serum proteins in the leopard seal, *Hydrurga leptonyx*, in Prydz Bay, eastern Antarctica and the coast of NSW, Australia. Comp Biochem Physiol B Biochem Mol Biol. (2005) 142:67–78. doi: 10.1016/j.cbpc.2005.05.016, PMID: 15993637

[63] Tatum LM Zaias J Mealey BK Cray C Bossart GD. Protein electrophoresis as a diagnostic and prognostic tool in raptor medicine. J Zoo Wildl Med. (2000) 31:497–502. doi: 10.1638/1042-7260(2000)031[0497:PEAADA]2.0.CO;2, PMID: 11428396

[64] Almberg ES Cross PC Dobson AP Smith DW Metz MC Stahler DR. Social living mitigates the costs of a chronic illness in a cooperative carnivore. Ecol Lett. (2015) 18:660–7. doi: 10.1111/ele.12444, PMID: 25983011 PMC4676290

[65] Kimura T. Comparative evaluation of acute phase proteins by C-reactive protein (CRP) and serum amyloid a (SAA) in nonhuman primates and feline carnivores. Anim Dis. (2022) 2:21. doi: 10.1186/s44149-022-00054-8

[66] Bornstein S Zakrisson G Thebo P. Clinical picture and antibody response to experimental *Sarcoptes scabiei* var. *vulpes* infection in red foxes (*Vulpes vulpes*). Acta Vet Scand. (1995) 36:509–19. doi: 10.1186/BF03547665, PMID: 8669378 PMC8095404

[67] Millan J Casais R Colomar V Bach E Prieto JM Velarde R. Experimental infection of wild-caught European rabbits (*Oryctolagus cuniculus*) with *Sarcoptes scabiei* from a naturally infected wild rabbit. Med Vet Entomol. (2013) 27:232–5. doi: 10.1111/j.1365-2915.2012.01035.x, PMID: 22958077

[68] Comolli JR Rivera S Wang C Cray C. Analysis of serum proteins in healthy giant pandas (*Ailuropoda melanoleuca*) under managed care. J Zoo Wildl Med. (2022) 53:442–7. doi: 10.1638/2020-0211, PMID: 35758586

[69] Niedringhaus KD Brown JD Ternent M Peltier SK Van Wick P Yabsley MJ. Serology as a tool to investigate sarcoptic mange in American black bears (*Ursus americanus*). J Wildl Dis. (2020) 56:350–8. doi: 10.7589/2019-04-086 31743065

[70] Bogan JE Jr. Analytical and clinical evaluation of two methods for measuring erythrocyte sedimentation rate in eastern indigo snakes (*Drymarchon couperi*). Animals. (2023) 13:464. doi: 10.3390/ani13030464, PMID: 36766352 PMC9913399

[71] Sheldon JD Johnson SP Hernandez JA Cray C Stacy NI. Acute-phase responses in health, malnourished, and *Otostrongylus* infected juvenile northern elephant seals (*Mirounga angustirostris*). J Zoo Wildl Med. (2017) 48:767–75. doi: 10.1638/2016-0267.1, PMID: 28920814

[72] Ceciliani F Giordano A Spagnolo V. The systemic reaction during inflammation: the acute-phase proteins. Protein Pept Lett. (2002) 9:211–23. doi: 10.2174/0929866023408779 12144517

[73] Pastor J Bach E Ráez-Bravo A López-Olvera JR Tvarijonaviciute A Granados JE. Method validation, reference values, and characterization of acute-phase protein responses to experimentally induced inflammation and bluetongue virus infection in the Iberian ibex. Vet Clin Pathol. (2019) 48:695–701. doi: 10.1111/vcp.12802, PMID: 31746492

[74] Gulati GL Hyland LJ Kocher W Schwarting R. Changes in automated complete blood cell count and differential leukocyte count results induced by storage of blood at room temperature. Arch Pathol Lab Med. (2002) 126:336–42. doi: 10.5858/2002-126-0336-CIACBC, PMID: 11860310

[75] Thorley RK Old JM. Distribution, abundance and threats to bare-nosed wombats (*Vombatus ursinus*). Aust Mammal. (2020) 42:249–56. doi: 10.1071/AM19035

[76] Donahoe SL Slapeta J Knowles G Obendorf D Peck S Phalen DN. Clinical and pathological features of toxoplasmosis in free-ranging common wombats (*Vombatus ursinus*) with multilocus genotyping of *toxoplasma gondii* type II-like strains. Parasitol Int. (2015) 64:148–53. doi: 10.1016/j.parint.2014.11.008 25463314

[77] Speight KN Whiteley PL Woolford L Duignan PJ Bacci B Lathe S. Outbreaks of sarcoptic mange in free-ranging koala populations in Victoria and South Australia: a case series. Aust Vet J. (2017) 95:244–9. doi: 10.1111/avj.12598, PMID: 28653387

