# Supplementary material for: Non-specific markers of inflammation in bare-nosed wombats (Vombatus ursinus) with sarcoptic mange
Source: Front Vet Sci. 2024 Jun 26;11:1403221. doi: 10.3389/fvets.2024.1403221 (PMC11240855; doi:10.3389/fvets.2024.1403221)
Supplement: Supplementary file 1 [file Table_2.docx]

**Supplementary Materials, S3**

Table 1. Sample collection date, assay results, and relevant clinical histories of zoo-housed bare-nosed (*Vombatus ursinus,* BNW) and southern hairy-nosed (*Lasiorhinus latifrons*, SHNW) wombats from a pilot study assessing cross-reactivity of wombat antibodies with commercially available immunoassays for serum amyloid A (SAA) and C-reactive protein (CRP).

| ID | Sample collection date | | Species | Vet-SAA~` | SAA-LZ~` | Multispecies SAA~^ | CRP~^+^ | Relevant clinical history |
| --- | --- | --- | --- | --- | --- | --- | --- | --- |
|  |  |  |  | (mg/L) | (mg/L) | (mg/L) | (mg/L) |  |
| 1 | 9/8/2003 | | SHNW | 0.8 |  | 5.2 | <0.10 | Annual health check* |
| 2 | 9/8/2010 | | SHNW | 0.8 |  | 40.4 | <0.10 | Annual health check* |
| 3 | 3/4/2017 | | BNW | 1.2 | <0.1 | 0.4 | 0.15 | Annual health check* |
| 4 | 9/8/2016 | | BNW | 1.3 | <0.1 | 0.2 | <0.10 | Annual health check* |
| 4 | 5/9/2017 | | BNW | 0.9 | <0.1 | 0.6 | <0.10 | Annual health check* |
| 5 | 16/12/2010 | | BNW | 0.5 | <0.1 | 0.2 | <0.10 | Unilateral uveitis |
| 5 | 25/6/2014 | | BNW | 0.5 | <0.1 | < 0.10 | <0.10 | Toxoplasmosis, neutrophilia, azotaemia |
| 6 | 18/3/2006 | | SHNW | 1.1 |  | < 0.10 | <0.10 | Dermatopathy |
| 2 | 15/2/2018 | | SHNW | 1.5 |  | 18.6 | <0.10 | Pancreatitis, peritonitis, cystitis (day of euthanasia) |
| 6 | 21/4/2015 | | SHNW | 0.5 |  | 4.4 | <0.10 | Oral squamous cell carcinoma (7 days prior to euthanasia) |
| 2 | 10/1/2018 | | SHNW | 1.1 |  | 2.8 | <0.10 | Pancreatitis (died one month later) |
| 4 | 26/9/2019 | | BNW | 1.0 | <0.1 | 3.0 | <0.10 | Nephrolithiasis, leucocytosis |
| 5 | 07/7/2014 | | BNW | <0.1 | <0.1 | < 0.10 | <0.20 | Interstitial nephritis, glomerulonephritis, aspiration pneumonia, toxoplasmosis |
| 3 | 21/6/2011 | | BNW | 1.3 | <0.1 | < 0.10 | <0.10 | Flea allergic dermatitis, secondary pyoderma |
|  | | ~ Reagents for these assays have not been validated for use in bare-nosed wombat (*Vombatus ursinus*)  ` Eiken Chemical Co., Japan  ^ Phase™ Range Multispecies SAA ELISA kit, Tridelta, Ireland  ^+^ Canine CRP Assay, Randox Laboratories, UK)  * Complete blood counts, serum biochemistry (which did not include serum protein electrophoretic fractions), and clinical examinations were within normal limits | | | | | | |
|  | |  | | | | | | |
