# Supplementary material for: Non-specific markers of inflammation in bare-nosed wombats (Vombatus ursinus) with sarcoptic mange
Source: Front Vet Sci. 2024 Jun 26;11:1403221. doi: 10.3389/fvets.2024.1403221 (PMC11240855; doi:10.3389/fvets.2024.1403221)
Supplement: Supplementary file 2 [file Data_Sheet_1.pdf]

Non-specific markers of inflammation

Shane A Richards

Model

This model looks for evidence of relation between non-specific markers of inflammation (NSMI) and mange scores. Specifically, we are interested in whether average mange scores are the best summary statistic to use as a predictor, or whether a NSMI could be better predicted based on (i) the highest mange score (i.e., on the 0-10 scale) observed, or (ii) the proportion of body segments showing signs of mange. For each individual, we have one or more NSMI and a set of J=14 body-segment scorings of mange $(x_i = x_{i,1}, \ldots x_{i,j})$, where each $x_{i,j}$ one is an integer ranging from 0 to 10 indicating mange severity. For each NSMI, the expected observed value for animal i, denoted $\mu(\mathbf{x}_i)$, is given by:

$$\mu(\mathbf{x}_i) = \alpha_0 \exp\left(\alpha_1 \sum_{j=1}^{J} (x_{i,j})^{\alpha_2}\right),$$

where α_0, α_1, and α_2 are parameters to be estimated. Variation in the observed NSMI values about the mean were assumed to be consistent with a log-normal distribution. Positive α_1 indicates the associated NSMI increases with higher mange scores, and vice-versa. α_2 describes how the J mange scores combine to quantify the mange status of an animal. For example, when $\alpha_2 = 1$ the average mange score describes mange status of an animal; however, when $\alpha_2 > 1$ mange status is more influenced by the highest mange score observed on the animal, and when $\alpha_2 < 1$ mange status is more influenced by the number of the 14 mange scores above zero.

Data

```
library(tidyverse)
library(cowplot)
library(rstan)
library(bayesplot)
library(tinytex)

rm(list = ls())

df_raw <- read_csv("app_all.csv") # read in the data

df_mange <- df_raw # save a copy of the data

# rename the state of the wombat
df_mange$HEALTH_1 <- factor(df_mange$HEALTH_1)
df_mange$state <- recode_factor(df_raw$HEALTH_1,
  "1" = "Healthy",
  "2" = "Mange (<25% body affected)",
  "3" = "Mange (>25% body affected)",
  "4" = "Unhealthy")

# the analysis excludes animals deemed unhealthy not due to mange
df_mange <- df_mange |>
  arrange(ESR) # sort lowest to highest ESR
df_mange$rank <- 1:nrow(df_mange) # add rankings

df_mange$ID <- factor(df_mange$ID) # add animal id as a factor

glimpse(df_mange) # show the fitted data
```

```
## Rows: 35
## Columns: 29
## $ ID        <fct> W-16 -07, BON4624, W-19-09, W-20-09, W-18-09, BON44559, BON2…
## $ HEALTH_1  <fct> 2, 1, 3, 1, 2, 3, 4, 3, 3, 3, 3, 4, 4, 3, 2, 2, 3, 4, 4, …
## $ LH        <dbl> 0, 0, 2, 0, 1, 5, 0, 7, 4, 10, 5, 10, 0, 0, 6, 3, 2, 4, 1, 1…
## $ LS        <dbl> 1, 0, 5, 0, 0, 3, 0, 3, 4, 10, 4, 10, 0, 0, 6, 0, 0, 4, 0, 0…
## $ LFL       <dbl> 0, 0, 3, 0, 0, 5, 0, 3, 5, 9, 6, 10, 0, 0, 8, 5, 1, 3, 1, 0,…
## $ LB        <dbl> 0, 0, 4, 1, 1, 4, 0, 6, 4, 10, 4, 0, 0, 0, 4, 0, 3, 4, 1, 1,…
## $ LF        <dbl> 2, 0, 6, 0, 2, 9, 0, 6, 10, 10, 5, 10, 0, 0, 8, 6, 5, 4, 0, …
## $ LR        <dbl> 0, 0, 0, 1, 0, 0, 0, 6, 3, 0, 0, 0, 0, 0, 2, 0, 2, 0, 0, 0, …
## $ LHL       <dbl> 1, 0, 2, 0, 0, 3, 0, 8, 5, 4, 6, 0, 0, 0, 3, 5, 3, 5, 0, 0, …
## $ RH        <dbl> 1, 0, 4, 0, 0, 4, 0, 4, 4, 10, 7, 0, 0, 0, 5, 3, 3, 8, 1, 1,…
## $ RS        <dbl> 2, 0, 2, 0, 0, 3, 0, 4, 4, 10, 7, 0, 0, 0, 5, 0, 1, 8, 0, 1,…
## $ RFL       <dbl> 1, 0, 2, 0, 1, 4, 0, 4, 5, 9, 7, 0, 0, 0, 8, 4, 3, 6, 1, 0, …
## $ RB        <dbl> 2, 0, 3, 0, 3, 3, 0, 7, 4, 9, 5, 0, 0, 0, 1, 0, 1, 6, 1, 0, …
## $ RF        <dbl> 3, 0, 6, 0, 4, 9, 0, 6, 10, 10, 9, 1, 0, 0, 8, 4, 4, 8, 0, 0…
## $ RR        <dbl> 2, 0, 0, 0, 2, 0, 0, 4, 3, 3, 1, 0, 0, 0, 1, 0, 2, 3, 0, 0, …
## $ RHL       <dbl> 2, 0, 2, 0, 2, 1, 0, 6, 5, 6, 4, 1, 0, 0, 2, 3, 4, 6, 0, 0, …
## $ AMS       <dbl> 1.21430000, 0.00000100, 2.92857143, 0.14290000, 1.14285714, …
## $ HAPTO_2   <dbl> 24.86, NA, NA, 0.75, NA, NA, NA, 1.86, NA, 10.67, 6.18, NA, …
## $ alb       <dbl> 376.0, NA, NA, 315.5, NA, NA, NA, 332.3, NA, 230.3, 322.3, N…
## $ a1        <dbl> 23.4, NA, NA, 36.0, NA, NA, NA, 24.2, NA, 48.8, 33.5, NA, NA…
## $ a2        <dbl> 43.8, NA, NA, 41.8, NA, NA, NA, 31.0, NA, 42.2, 40.2, NA, NA…
## $ b         <dbl> 109.5, NA, NA, 103.8, NA, NA, NA, 96.7, NA, 106.9, 106.5, NA…
## $ y         <dbl> 177.4, NA, NA, 82.9, NA, NA, NA, 135.8, NA, 231.7, 167.5, NA…
## $ ESR       <dbl> 2.10, 2.20, 3.20, 3.30, 7.70, 8.25, 8.40, 8.90, 9.17, 10.45,…
## $ HEALTHAMS <dbl> 2, 1, 3, 1, 2, 3, 4, 3, 3, 3, 3, 4, 4, 3, 2, 2, 3, 4, 4, …
## $ HEALTH_N  <dbl> 2, 1, 3, 1, 2, 3, 3, 3, 3, 3, 3, 3, 3, 3, 2, 2, 3, NA, NA…
## $ ...27     <chr> NA, NA, NA, NA, NA, NA, NA, NA, NA, NA, NA, NA, NA, NA, NA, …
## $ state     <fct> Mange (<25% body affected), Healthy, Mange (>25% body affect…
## $ rank      <int> 1, 2, 3, 4, 5, 6, 7, 8, 9, 10, 11, 12, 13, 14, 15, 16, 17, 1…
```

```
#| message: false
#| warning: false
#| fig-width: 7.5
#| fig-align: center
#| fig-asp: 0.5
#| fig-cap: Relationship between average mange scofre (AMS) and seven measured responses.
#| label: fig-responses

df_mange$AMS_obs <- 0.0
for (i in 1:nrow(df_mange)) {
    df_mange$AMS_obs[i] <- sum(df_mange[i,3:16])/14
}

df_plot_1 <- df_mange %>%
  select("AMS_obs", "state", "alb", "a1", "a2", "b", "y", "HAPTO_2", "ESR") %>%
  pivot_longer(names_to = "NSMI", values_to = "Value", 3:9)

df_plot_1$state <- factor(df_plot_1$state, levels = c("Unhealthy", "Healthy", "Mange (<25% body affected)", "Mang
e (>25% body affected)"))

ggplot(filter(df_plot_1, state != "U")) +
  geom_point(aes(y = Value, x = AMS_obs, color = state)) +
  facet_wrap( ~ NSMI, scale = "free_y", ncol = 4) +
    labs(x = "Average mange score (AMS)", y = "Response", color = "State") +
    scale_color_manual(values = c("black", "#74c476", "#feb24c", "#e31a1c")) +
  theme_bw() +
  theme(panel.grid = element_blank())
```

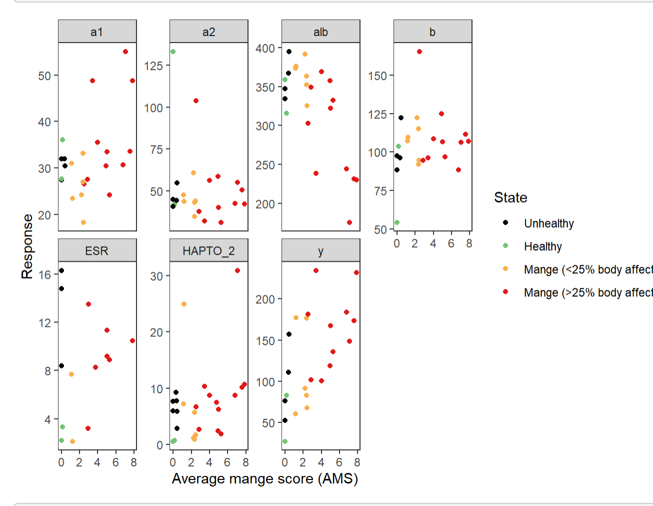


```
NSMIs <- names(df_mange)[18:24] # possible responses to include below
NSMIs
```

```
## [1] "HAPTO_2" "alb"     "a1"      "a2"      "b"       "y"       "ESR"
```

```
df_fits <- tibble(NSMI = NSMIs,
    a0 = 0.0, a0l = 0.0, a0u = 0.0, a1 = 0.0, a1l = 0.0, a1u = 0.0,
  a2 = 0.0, a2l = 0.0, a2u = 0.0, cred = 0.0)

df_plot <- NULL

rw <- 0
for (y_txt in NSMIs) {
    cat(y_txt)
    rw <- rw + 1
  df_fit <- df_mange %>%
    select(ID, HEALTH_1, state,
      LH, LS, LFL, LB, LF, LR, LHL, RH, RS, RFL, RB, RF, RR, RHL, AMS,
      Response = y_txt) %>%
    filter(state != "Unhealthy") |>
    na.omit()

  df_MSs <- df_fit %>%
    dplyr::select(-any_of(c("ID", "HEALTH_1", "state", "AMS", "Response")))

  m_mange <- as.matrix(df_MSs)

  df_fit$AMS <- apply(m_mange, MARGIN = 1, FUN = mean)

  v_y <- as.numeric(df_fit$Response)
  x   <- m_mange
  s   <- rep(1:7,2) # segments (7 of them, repeated left/right)

  I <- nrow(m_mange) # number of animals
  J <- ncol(m_mange) # number of body segments (whole body)

  # create the list of data for stan
  stan_dat <- list(
    I = I,   # number of animals
    J = J,   # number of body segments
    x = x,   # mange scores
    y = v_y) # segment (1-7)

  # fit the model!
  model_params <- c("alpha_0", "alpha_1", "alpha_2", "sigma")
  fit_continuous <- stan(file = 'Model_continuous.stan', data = stan_dat,
    iter = 2000, warmup = 1000, chains = 1, refresh = 2000)

  l_param_continuous <- rstan::extract(fit_continuous, pars = model_params)

  sigma   <- l_param_continuous$sigma
  alpha_0 <- l_param_continuous$alpha_0
  alpha_1 <- l_param_continuous$alpha_1
  alpha_2 <- l_param_continuous$alpha_2

  df_fits$a0[rw]  <- median(alpha_0)
  df_fits$a0l[rw] <- quantile(alpha_0, probs = 0.025)
  df_fits$a0u[rw] <- quantile(alpha_0, probs = 0.975)

  df_fits$a1[rw]  <- median(alpha_1)
  df_fits$a1l[rw] <- quantile(alpha_1, probs = 0.025)
  df_fits$a1u[rw] <- quantile(alpha_1, probs = 0.975)

  df_fits$a2[rw]  <- median(alpha_2)
  df_fits$a2l[rw] <- quantile(alpha_2, probs = 0.025)
  df_fits$a2u[rw] <- quantile(alpha_2, probs = 0.975)

  df_fits$cred[rw] <- mean(alpha_1 > 0.0)

  df_fits$a0[rw]  <- round(df_fits$a0[rw], 2)
  df_fits$a0l[rw] <- round(df_fits$a0l[rw], 2)
  df_fits$a0u[rw] <- round(df_fits$a0u[rw], 2)

  df_fits$a1[rw]  <- round(df_fits$a1[rw], 3)
  df_fits$a1l[rw] <- round(df_fits$a1l[rw], 3)
  df_fits$a1u[rw] <- round(df_fits$a1u[rw], 3)

  df_fits$a2[rw]  <- round(df_fits$a2[rw], 2)
  df_fits$a2l[rw] <- round(df_fits$a2l[rw], 2)
  df_fits$a2u[rw] <- round(df_fits$a2u[rw], 2)

  df_fits$cred[rw] <- round(df_fits$cred[rw], 3)

  mcmc_hist(fit_continuous, pars = model_params)

  # fit the model!
  model_params <- c("alpha_0", "alpha_1", "sigma")
  fit_continuous <- stan(file = 'Model_continuous_AMS.stan',
    data = stan_dat, iter = 4000, warmup = 2000, chains = 1, refresh = 2000)

  l_param_continuous <- rstan::extract(fit_continuous, pars = model_params)

  sigma   <- l_param_continuous$sigma
  alpha_0 <- l_param_continuous$alpha_0
  alpha_1 <- l_param_continuous$alpha_1

  REPS <- length(sigma)
  y_predict <- rep(0, REPS)

  SMS <- apply(m_mange, 1, sum)

  df_fit$SMS <- SMS

  # scaled mange scores used for predictions
  SMSs <- seq(from = min(SMS), to = max(SMS), length.out = 50)

  # create a data frame of predictions
  df_mu_continuous <- tibble(NSMI = y_txt, x = SMSs,
    low95 = 0.0, mdn = 0.0, upp95 = 0.0)

  df_fit_raw <- tibble(NSMI = y_txt, y_obs = stan_dat$y,
    AMS = df_fit$AMS)

  for (i in 1:nrow(df_mu_continuous)) {
    probs <- quantile(alpha_0 * exp(alpha_1*df_mu_continuous$x[i]),
      probs = c(0.025,0.5,0.975))
    df_mu_continuous$low95[i] <- probs[1]
    df_mu_continuous$mdn[i]   <- probs[2]
    df_mu_continuous$upp95[i] <- probs[3]
  }

  if (is.null(df_plot)) {
    df_plot <- df_mu_continuous
    df_plot_raw <- df_fit_raw
  } else {
    df_plot <- rbind(df_plot, df_mu_continuous)
    df_plot_raw <- rbind(df_plot_raw, df_fit_raw)
  }
}
```

```
## HAPTO_2
## SAMPLING FOR MODEL 'anon_model' NOW (CHAIN 1).
## Chain 1:
## Chain 1: Gradient evaluation took 6e-05 seconds
## Chain 1: 1000 transitions using 10 leapfrog steps per transition would take 0.6 seconds.
## Chain 1: Adjust your expectations accordingly!
## Chain 1:
## Chain 1:
## Chain 1: Iteration:    1 / 2000 [  0%]  (Warmup)
## Chain 1: Iteration: 1001 / 2000 [ 50%]  (Sampling)
## Chain 1: Iteration: 2000 / 2000 [100%]  (Sampling)
## Chain 1:
## Chain 1:  Elapsed Time: 0.901 seconds (Warm-up)
## Chain 1:                0.87 seconds (Sampling)
## Chain 1:                1.771 seconds (Total)
## Chain 1:
##
## SAMPLING FOR MODEL 'anon_model' NOW (CHAIN 1).
## Chain 1:
## Chain 1: Gradient evaluation took 2.6e-05 seconds
## Chain 1: 1000 transitions using 10 leapfrog steps per transition would take 0.26 seconds.
## Chain 1: Adjust your expectations accordingly!
## Chain 1:
## Chain 1:
## Chain 1: Iteration:    1 / 4000 [  0%]  (Warmup)
## Chain 1: Iteration: 2000 / 4000 [ 50%]  (Warmup)
## Chain 1: Iteration: 2001 / 4000 [ 50%]  (Sampling)
## Chain 1: Iteration: 4000 / 4000 [100%]  (Sampling)
## Chain 1:
## Chain 1:  Elapsed Time: 0.246 seconds (Warm-up)
## Chain 1:                0.245 seconds (Sampling)
## Chain 1:                0.491 seconds (Total)
## Chain 1:
## alb
## SAMPLING FOR MODEL 'anon_model' NOW (CHAIN 1).
## Chain 1:
## Chain 1: Gradient evaluation took 5e-05 seconds
## Chain 1: 1000 transitions using 10 leapfrog steps per transition would take 0.5 seconds.
## Chain 1: Adjust your expectations accordingly!
## Chain 1:
## Chain 1:
## Chain 1: Iteration:    1 / 2000 [  0%]  (Warmup)
## Chain 1: Iteration: 1001 / 2000 [ 50%]  (Sampling)
## Chain 1: Iteration: 2000 / 2000 [100%]  (Sampling)
## Chain 1:
## Chain 1:  Elapsed Time: 0.552 seconds (Warm-up)
## Chain 1:                0.539 seconds (Sampling)
## Chain 1:                1.091 seconds (Total)
## Chain 1:
##
## SAMPLING FOR MODEL 'anon_model' NOW (CHAIN 1).
## Chain 1:
## Chain 1: Gradient evaluation took 2.7e-05 seconds
## Chain 1: 1000 transitions using 10 leapfrog steps per transition would take 0.27 seconds.
## Chain 1: Adjust your expectations accordingly!
## Chain 1:
## Chain 1:
## Chain 1: Iteration:    1 / 4000 [  0%]  (Warmup)
## Chain 1: Iteration: 2000 / 4000 [ 50%]  (Warmup)
## Chain 1: Iteration: 2001 / 4000 [ 50%]  (Sampling)
## Chain 1: Iteration: 4000 / 4000 [100%]  (Sampling)
## Chain 1:
## Chain 1:  Elapsed Time: 0.212 seconds (Warm-up)
## Chain 1:                0.171 seconds (Sampling)
## Chain 1:                0.383 seconds (Total)
## Chain 1:
## a1
## SAMPLING FOR MODEL 'anon_model' NOW (CHAIN 1).
## Chain 1:
## Chain 1: Gradient evaluation took 5.7e-05 seconds
## Chain 1: 1000 transitions using 10 leapfrog steps per transition would take 0.57 seconds.
## Chain 1: Adjust your expectations accordingly!
## Chain 1:
## Chain 1:
## Chain 1: Iteration:    1 / 2000 [  0%]  (Warmup)
## Chain 1: Iteration: 1001 / 2000 [ 50%]  (Sampling)
## Chain 1: Iteration: 2000 / 2000 [100%]  (Sampling)
## Chain 1:
## Chain 1:  Elapsed Time: 0.793 seconds (Warm-up)
## Chain 1:                0.552 seconds (Sampling)
## Chain 1:                1.345 seconds (Total)
## Chain 1:
##
## SAMPLING FOR MODEL 'anon_model' NOW (CHAIN 1).
## Chain 1:
## Chain 1: Gradient evaluation took 4e-05 seconds
## Chain 1: 1000 transitions using 10 leapfrog steps per transition would take 0.4 seconds.
## Chain 1: Adjust your expectations accordingly!
## Chain 1:
## Chain 1:
## Chain 1: Iteration:    1 / 4000 [  0%]  (Warmup)
## Chain 1: Iteration: 2000 / 4000 [ 50%]  (Warmup)
## Chain 1: Iteration: 2001 / 4000 [ 50%]  (Sampling)
## Chain 1: Iteration: 4000 / 4000 [100%]  (Sampling)
## Chain 1:
## Chain 1:  Elapsed Time: 0.259 seconds (Warm-up)
## Chain 1:                0.222 seconds (Sampling)
## Chain 1:                0.481 seconds (Total)
## Chain 1:
## a2
## SAMPLING FOR MODEL 'anon_model' NOW (CHAIN 1).
## Chain 1:
## Chain 1: Gradient evaluation took 7.6e-05 seconds
## Chain 1: 1000 transitions using 10 leapfrog steps per transition would take 0.76 seconds.
## Chain 1: Adjust your expectations accordingly!
## Chain 1:
## Chain 1:
## Chain 1: Iteration:    1 / 2000 [  0%]  (Warmup)
## Chain 1: Iteration: 1001 / 2000 [ 50%]  (Sampling)
## Chain 1: Iteration: 2000 / 2000 [100%]  (Sampling)
## Chain 1:
## Chain 1:  Elapsed Time: 0.749 seconds (Warm-up)
## Chain 1:                0.682 seconds (Sampling)
## Chain 1:                1.431 seconds (Total)
## Chain 1:
##
## SAMPLING FOR MODEL 'anon_model' NOW (CHAIN 1).
## Chain 1:
## Chain 1: Gradient evaluation took 4.1e-05 seconds
## Chain 1: 1000 transitions using 10 leapfrog steps per transition would take 0.41 seconds.
## Chain 1: Adjust your expectations accordingly!
## Chain 1:
## Chain 1:
## Chain 1: Iteration:    1 / 4000 [  0%]  (Warmup)
## Chain 1: Iteration: 2000 / 4000 [ 50%]  (Warmup)
## Chain 1: Iteration: 2001 / 4000 [ 50%]  (Sampling)
## Chain 1: Iteration: 4000 / 4000 [100%]  (Sampling)
## Chain 1:
## Chain 1:  Elapsed Time: 0.443 seconds (Warm-up)
## Chain 1:                0.363 seconds (Sampling)
## Chain 1:                0.806 seconds (Total)
## Chain 1:
## b
## SAMPLING FOR MODEL 'anon_model' NOW (CHAIN 1).
## Chain 1:
## Chain 1: Gradient evaluation took 5.4e-05 seconds
## Chain 1: 1000 transitions using 10 leapfrog steps per transition would take 0.54 seconds.
## Chain 1: Adjust your expectations accordingly!
## Chain 1:
## Chain 1:
## Chain 1: Iteration:    1 / 2000 [  0%]  (Warmup)
## Chain 1: Iteration: 1001 / 2000 [ 50%]  (Sampling)
## Chain 1: Iteration: 2000 / 2000 [100%]  (Sampling)
## Chain 1:
## Chain 1:  Elapsed Time: 0.905 seconds (Warm-up)
## Chain 1:                0.892 seconds (Sampling)
## Chain 1:                1.797 seconds (Total)
## Chain 1:
##
## SAMPLING FOR MODEL 'anon_model' NOW (CHAIN 1).
## Chain 1:
## Chain 1: Gradient evaluation took 2.6e-05 seconds
## Chain 1: 1000 transitions using 10 leapfrog steps per transition would take 0.26 seconds.
## Chain 1: Adjust your expectations accordingly!
## Chain 1:
## Chain 1:
## Chain 1: Iteration:    1 / 4000 [  0%]  (Warmup)
## Chain 1: Iteration: 2000 / 4000 [ 50%]  (Warmup)
## Chain 1: Iteration: 2001 / 4000 [ 50%]  (Sampling)
## Chain 1: Iteration: 4000 / 4000 [100%]  (Sampling)
## Chain 1:
## Chain 1:  Elapsed Time: 0.279 seconds (Warm-up)
## Chain 1:                0.282 seconds (Sampling)
## Chain 1:                0.561 seconds (Total)
## Chain 1:
## y
## SAMPLING FOR MODEL 'anon_model' NOW (CHAIN 1).
## Chain 1:
## Chain 1: Gradient evaluation took 5.1e-05 seconds
## Chain 1: 1000 transitions using 10 leapfrog steps per transition would take 0.51 seconds.
## Chain 1: Adjust your expectations accordingly!
## Chain 1:
## Chain 1:
## Chain 1: Iteration:    1 / 2000 [  0%]  (Warmup)
## Chain 1: Iteration: 1001 / 2000 [ 50%]  (Sampling)
## Chain 1: Iteration: 2000 / 2000 [100%]  (Sampling)
## Chain 1:
## Chain 1:  Elapsed Time: 0.85 seconds (Warm-up)
## Chain 1:                1.023 seconds (Sampling)
## Chain 1:                1.873 seconds (Total)
## Chain 1:
##
## SAMPLING FOR MODEL 'anon_model' NOW (CHAIN 1).
## Chain 1:
## Chain 1: Gradient evaluation took 6.7e-05 seconds
## Chain 1: 1000 transitions using 10 leapfrog steps per transition would take 0.67 seconds.
## Chain 1: Adjust your expectations accordingly!
## Chain 1:
## Chain 1:
## Chain 1: Iteration:    1 / 4000 [  0%]  (Warmup)
## Chain 1: Iteration: 2000 / 4000 [ 50%]  (Warmup)
## Chain 1: Iteration: 2001 / 4000 [ 50%]  (Sampling)
## Chain 1: Iteration: 4000 / 4000 [100%]  (Sampling)
## Chain 1:
## Chain 1:  Elapsed Time: 0.73 seconds (Warm-up)
## Chain 1:                0.562 seconds (Sampling)
## Chain 1:                1.292 seconds (Total)
## Chain 1:
## ESR
## SAMPLING FOR MODEL 'anon_model' NOW (CHAIN 1).
## Chain 1:
## Chain 1: Gradient evaluation took 5.5e-05 seconds
## Chain 1: 1000 transitions using 10 leapfrog steps per transition would take 0.55 seconds.
## Chain 1: Adjust your expectations accordingly!
## Chain 1:
## Chain 1:
## Chain 1: Iteration:    1 / 2000 [  0%]  (Warmup)
## Chain 1: Iteration: 1001 / 2000 [ 50%]  (Sampling)
## Chain 1: Iteration: 2000 / 2000 [100%]  (Sampling)
## Chain 1:
## Chain 1:  Elapsed Time: 0.66 seconds (Warm-up)
## Chain 1:                0.392 seconds (Sampling)
## Chain 1:                1.052 seconds (Total)
## Chain 1:
##
## SAMPLING FOR MODEL 'anon_model' NOW (CHAIN 1).
## Chain 1:
## Chain 1: Gradient evaluation took 3.6e-05 seconds
## Chain 1: 1000 transitions using 10 leapfrog steps per transition would take 0.36 seconds.
## Chain 1: Adjust your expectations accordingly!
## Chain 1:
## Chain 1:
## Chain 1: Iteration:    1 / 4000 [  0%]  (Warmup)
## Chain 1: Iteration: 2000 / 4000 [ 50%]  (Warmup)
## Chain 1: Iteration: 2001 / 4000 [ 50%]  (Sampling)
## Chain 1: Iteration: 4000 / 4000 [100%]  (Sampling)
## Chain 1:
## Chain 1:  Elapsed Time: 0.305 seconds (Warm-up)
## Chain 1:                0.311 seconds (Sampling)
## Chain 1:                0.616 seconds (Total)
## Chain 1:
```

```
df_fits
```

NSMI <chr>	a0 <dbl>	a0l <dbl>	a0u <dbl>	a1 <dbl>	a1l <dbl>	a1u <dbl>	a2 <dbl>	a2l <dbl>	a2u <dbl>
HAPTO_2	1.43	0.51	4.20	0.042	0.005	0.119	0.51	0.19	1.17
alb	142.38	114.19	148.29	0.042	0.021	0.062	0.23	0.14	0.55
a1	25.80	19.30	34.07	0.006	-0.003	0.027	0.54	0.15	1.49
a2	68.99	43.49	106.78	-0.021	-0.052	0.003	0.29	0.14	0.93
b	94.38	73.25	116.96	0.004	-0.006	0.020	0.40	0.16	1.15
y	61.12	35.29	95.27	0.032	0.006	0.075	0.43	0.15	1.07
ESR	3.05	1.30	6.36	0.027	0.001	0.082	0.61	0.16	1.08

7 rows | 1-10 of 11 columns
